# UV-DDB stimulates the activity of SMUG1 during base excision repair of 5-hydroxymethyl-2'-deoxyuridine moieties

**DOI:** 10.1093/nar/gkad206

**Published:** 2023-03-27

**Authors:** Sunbok Jang, Sripriya J Raja, Vera Roginskaya, Matthew A Schaich, Simon C Watkins, Bennett Van Houten

**Affiliations:** UPMC Hillman Cancer Center, University of Pittsburgh, Pittsburgh, PA 15213, USA; College of Pharmacy, Graduate School of Pharmaceutical Sciences, Ewha Womans University, Seoul 03760, Republic of Korea; Department of Pharmacology and Chemical Biology, School of Medicine, University of Pittsburgh, Pittsburgh, PA 15213, USA; UPMC Hillman Cancer Center, University of Pittsburgh, Pittsburgh, PA 15213, USA; Molecular Pharmacology Graduate Program, School of Medicine, University of Pittsburgh, Pittsburgh, PA 15213, USA; UPMC Hillman Cancer Center, University of Pittsburgh, Pittsburgh, PA 15213, USA; Department of Pharmacology and Chemical Biology, School of Medicine, University of Pittsburgh, Pittsburgh, PA 15213, USA; UPMC Hillman Cancer Center, University of Pittsburgh, Pittsburgh, PA 15213, USA; Department of Pharmacology and Chemical Biology, School of Medicine, University of Pittsburgh, Pittsburgh, PA 15213, USA; Center for Biologic Imaging, University of Pittsburgh, Pittsburgh, PA 15261, USA; UPMC Hillman Cancer Center, University of Pittsburgh, Pittsburgh, PA 15213, USA; Molecular Pharmacology Graduate Program, School of Medicine, University of Pittsburgh, Pittsburgh, PA 15213, USA; Department of Pharmacology and Chemical Biology, School of Medicine, University of Pittsburgh, Pittsburgh, PA 15213, USA

## Abstract

UV-damaged DNA-binding protein (UV-DDB) is a heterodimeric protein, consisting of DDB1 and DDB2 subunits, that works to recognize DNA lesions induced by UV damage during global genome nucleotide excision repair (GG-NER). Our laboratory previously discovered a non-canonical role for UV-DDB in the processing of 8-oxoG, by stimulating 8-oxoG glycosylase, OGG1, activity 3-fold, MUTYH activity 4-5-fold, and APE1 (apurinic/apyrimidinic endonuclease 1) activity 8-fold. 5-hydroxymethyl-deoxyuridine (5-hmdU) is an important oxidation product of thymidine which is removed by single-strand selective monofunctional DNA glycosylase (SMUG1). Biochemical experiments with purified proteins indicated that UV-DDB stimulates the excision activity of SMUG1 on several substrates by 4-5-fold. Electrophoretic mobility shift assays indicated that UV-DDB displaced SMUG1 from abasic site products. Single-molecule analysis revealed that UV-DDB decreases the half-life of SMUG1 on DNA by ∼8-fold. Immunofluorescence experiments demonstrated that cellular treatment with 5-hmdU (5 μM for 15 min), which is incorporated into DNA during replication, produces discrete foci of DDB2-mCherry, which co-localize with SMUG1-GFP. Proximity ligation assays supported a transient interaction between SMUG1 and DDB2 in cells. Poly(ADP)-ribose accumulated after 5-hmdU treatment, which was abrogated with SMUG1 and DDB2 knockdown. These data support a novel role for UV-DDB in the processing of the oxidized base, 5-hmdU.

## INTRODUCTION

Endogenous products of metabolism, as well as environmental toxicants, create DNA modifications that compromise genomic stability (1). Base excision repair (BER) works to remove DNA damage created through alkylation, oxidation, deamination and replication errors (2). Mammalian BER is initiated by one of 11 glycosylases that recognize specific base modifications (2,3). One common form of base damage is uracil which results from the deamination of cytosine, and the DNA polymerase-catalyzed incorporation of dUTP in place of dTTP during replication. Cytosine deamination has been estimated to occur 60–500 times per genome per day (4). Measurements of dUTP incorporation into mammalian DNA vary but have been estimated to occur at steady-state levels of 70–200 per day (1,5).

Biochemical studies have shown that uracil DNA glycosylase (UNG), single-strand selective monofunctional DNA glycosylase (SMUG1), thymine DNA glycosylase (TDG) and methyl-CpG binding domain 4 DNA glycosylase (MBD4), all work on substrates containing uracil and modified uracil moieties in dsDNA (4). SMUG1 and UNG are primarily responsible for the removal of uracil from U:A and U:G base pairs, through the short-patch BER pathway (6,7). U:G mispairs, created from the deamination of cytosine, lead to C to T transition mutations, a common mutational signature of several human cancers (8,9).

Another important uracil moiety found in DNA is 5-hydroxymethyl 2-deoxyuridine (5-hmdU), which is created as a result of the direct oxidation of thymidine by the enzymatic activity of tet methylcytosine dioxygenase (TET1) (10,11), ionizing radiation (12), hydrogen peroxide or oxidant stress (13,14). Recently, it was demonstrated that the level of 5-hmdU in various cultured human cells and mammalian tissues is roughly 3–8 modifications per million nucleotides (10). It has also been proposed that deamination of the product of oxidative demethylation of 5-methylcytosine, 5-hydroxylmethylcytosine, can produce 5-hmdU, causing a 5-hmdU:G mispair (15,16). SMUG1 repairs 5-hmdU and other DNA-pyrimidine oxidation products such as 5-formyldeoxyuridine (fdU) from DNA (17). The mouse and human SMUG1 can work efficiently on lesions embedded in either single-stranded or double-stranded DNA (17,18). SMUG1 has been found to have weak excision activity in the removal of fluorodeoxyuridine (FdU) and fdU moieties from DNA (4,18).

An and coworkers found that *Ung^−/−^* mouse embryonic fibroblasts (MEF) in which SMUG1 had been depleted through siRNA, showed increased sensitivity to both IR and FdU (19,20). Interestingly, MEF cells deficient in *Smug1^−/−^* are protected from the cytotoxic effects of administration of the nucleoside, 5-hmdU, which is readily converted to the triphosphate and incorporated into actively growing cells (16). They also showed that nuclear extracts from the brain or liver of *Smug1*^−/−^ mice were deficient in the removal of hmdU:G and that UNG could not compensate for this activity in extracts. Finally, by creating triple *Ung*^−/−^*Smug1*^−/−^*Msh2*^−/−^ KO mice, it was shown that the loss of these three genes resulted in an extremely high rate of tumors and death in mice by 150 days. These data reveal that UNG and SMUG1 collaborate to limit 5-hmdU and mutagenic lesions in the genome (21).

SMUG1, like other monofunctional glycosylases, is product inhibited binding more avidly to the abasic site product than the target lesion, 5-hmdU (7). This product inhibition causes slow enzymatic turnover of SMUG1 (22). Furthermore, SMUG1 has difficulties removing uracil from nucleosomes, the efficiency decreases 3 to 9-fold compared to naked DNA (23). Subsequent analysis of defined sites within a 601 nucleosome sequence showed that SMUG1 activity was highly suppressed regardless of the position of the uracil on the nucleosome (24). Due to SMUG1 product inhibition, it has been proposed that APE1 or XPC may work to help turnover SMUG1 from abasic sites (22,25). We have previously shown that UV-damaged DNA-binding protein, UV-DDB, a heterodimeric protein consisting of DDB1 (127 kDa) and DDB2 (48 kDa) subunits, plays a non-canonical role in the processing of 8-oxoG by stimulating 8-oxoG glycosylase (OGG1), MUTYH, apurinic/apyrimidinic endonuclease 1, APE1 and alkyladenine glycosylase (AAG), also known as N-methylpurine DNA glycosylase (MPG) activities through a facilitated dissociation mechanism by direct displacement of these proteins from abasic sites (26–28).

In this present study, we have investigated whether UV-DDB can help stimulate SMUG1, and thus act as the first responder for 5-hmdU damage sensing and repair. We find that UV-DDB stimulates uracil and 5-hmdU excision by SMUG1 by 4-5-fold and helps dissociate SMUG1 from abasic sites. Single-molecule studies showed that UV-DDB decreased the half-life of SMUG1 on abasic sites. Cellular experiments indicate that SMUG1 and UV-DDB form transient intermediates after 5-hmdU treatment. Moreover, we show that SMUG1 and DDB2 work together to limit 5-hmdU toxicity in cells. Together these data provide insights into the molecular mechanisms for uracil excision in cells.

## MATERIALS AND METHODS

### Expression and purification of recombinant UV-DDB and SMUG1

Recombinant full-length UV-DDB (DDB1–DDB2 heterodimer) was expressed in Sf9 cells coinfected with recombinant baculovirus of DDB1-6xHis and DDB2-Flag, as performed previously (26,27). Briefly, DDB1-6xHis and DDB2-Flag were purified using a 5 ml His-Trap HP column pre-charged with Ni^2+^ (GE Healthcare) and anti-FLAG M2 affinity gel (Sigma). The pooled anti-FLAG eluates were size fractionated on a HiLoad 16/60 Superdex 200 column (Amersham Pharmacia) in UV-DDB storage buffer (50 mM HEPES, pH 7.5, 200 mM KCl, 1 mM EDTA, 0.5 mM PMSF, 2 mM DTT, 10% glycerol and 0.02% sodium azide). Purified fractions of the DDB1–DDB2 complex from the Superdex200 were aliquoted and flash-frozen with liquid nitrogen and stored at −80°C. SMUG1 WT was purchased from NOVUS (Saint Charles, MO).

### Cell lines

U2OS cells were cultured in 5% CO_2_ in Dulbecco's modified Eagle's medium (DMEM) supplemented with 4.5 g/l glucose, 10% fetal bovine serum (Gibco), 5% penicillin/streptavidin (Life Technologies). To obtain transient expression of SMUG1 and DDB2, U2OS cells were transfected with 500 ng each of SMUG1-GFP (Origene Cat# RG212141) and DDB2-mCherry (Gene Universal) using the lipofectamine 2000 reagent and protocol (Thermo Fisher Cat# 11668019). DDB2-mCherry was constructed by Gene Universal Inc., as described in (29). Briefly, DDB2-mCherry was constructed by removing the mNeonGreen sequence through restriction and cloning in the mCherry sequence.

### DNA substrate preparation

#### 37 bp DNA duplexes for excision assay and native gel experiment

The following oligonucleotides sequences (X = 5-hmdU and Z = tetrahydrofuran) were used:

UD37-top: 5 ‘-CCG AGT CAT TCC TGC AGC GAG TCC ATG GGA GTC AAA T-3’-6FAM

UD37-bottom: 5′-ATT TGA CTC CCA TGG ACT CGC TGC AGG AAT GAC TCG G-3′

dU:dA or dU:dG-top: 5′-CCG AGT CAT TCC TGC AGC GAG **U**CC ATG GGA GTC AAA T-3′-6FAM

5-hmdU:dA or 5-hmdU:dG-top: 5′-CCG AGT CAT TCC TGC AGC GAG **X**CC ATG GGA GTC AAA T-3′-6FAM

dU:dA or 5-hmdU:dA-bottom: 5′-A TTT GAC TCC CAT GG**A** CTC GCT GCA GGA ATG ACT CGG-3′

dU:dG or 5-hmdU:dG-bottom: 5′-A TTT GAC TCC CAT GG**G** CTC GCT GCA GGA ATG ACT CGG-3′

THF37-top: 5′-CCG AGT CAT TCC TGC AGC G**Z**G TCC ATG GGA GTC AAA T-3′-6FAM

THF37-bottom: 5′-ATT TGA CTC CCA TGG ACT CGC TGC AGG AAT GAC TCG G-3′Double strand UD37, dU:dA37, dU:dG37, 5-hmdU:dA37, 5-hmdU:dG37 and THF37 were prepared by annealing each top and bottom strand (all purchased from IDT, USA). Annealing reactions were done at 95°C for 5 min in 10 mM Tris–HCl, pH 8.0, and 100 mM KCl and then cooled to room temperature (RT) slowly for 4 hr by turning off the heating device.

#### Defined lesion plasmids

Plasmids containing single site-specific THF adducts were prepared as described previously (26). Briefly, purified pSCW01 plasmids were nicked by *Nt.BstNBI* to create a 37-base gap. A 37-mer containing a single abasic site (THF37-top, above) was annealed into this gap and the backbone was sealed with T4 DNA ligase. The THF arrays were prepared using the defined lesion plasmid described above. Lesion-containing pSCW01 was linearized via restriction digest by *XhoI* (NEB) and then incubated with T4 DNA ligase (NEB) to achieve long (> 40 kbp) tandemly ligated products with one THF site every 2 kb.

### Equilibrium dissociation constant determination from electrophoretic mobility shift assay (EMSA)

To determine the equilibrium dissociation constants of UV-DDB binding to various DNA lesions we conducted electrophoretic mobility shift assays (EMSA) with fluorescein-modified DNA substrates. DNA substrates, held constant at 8 nM, were mixed with increasing amounts of UV-DDB and incubated for 20 min at room temperature (RT) in reaction buffer (20 mM HEPES, pH 7.5, 150 mM NaCl, 2 mM DTT, 5% glycerol, 0.5 mg/ml BSA). A 5 μl aliquot of each reaction was loaded on a 5% polyacrylamide (37.5:1, acrylamide: bis) native gel, in duplicate, and run at 100 V for 50 min at 4°C in 1/2× TBE. 1.5 mM MgCl_2_ was added into the reaction buffer, gels, and running buffer when EMSA was conducted to see the effect of magnesium on increasing specificity. Gels were imaged using a laser scanner for fluorescence (Typhoon, Amersham).

Gel images were quantified by measuring the signal intensity of each band (ImageJ, NIH). The percentage of DNA bound was determined by dividing the intensity of the shifted (‘bound’) DNA by the sum of all bands in a lane. Background signals from blank regions of the gel were subtracted from the signal intensities obtained from bands. These values were plotted against UV-DDB concentration, and the data were fit to the following equation via nonlinear regression in GraphPad Prism:


$$\begin{eqnarray*}\% \ {\rm DNA}\ {\rm bound}\ = {\rm{\ }}100{\rm{\ }} \times {\rm{\ }}\frac{{\left( {D + P + {K}_{\rm d}} \right) - \sqrt {{{\left( {D + P + {K}_{\rm d}} \right)}}^2 - 4DP} }}{{2D}}\end{eqnarray*}$$

where *K*_d_ is the equilibrium dissociation constant, *P* is the total protein concentration, and *D* is the total DNA concentration, all in units M. This model was chosen because our experimental conditions required that the DNA concentration is in the same molar range as the *K*_d_ (30). For our fitting, we constrained the DNA concentration at the expected 8 nM, and the maximum plateau as 100% (completely bound).

### SMUG1 excision assay

Reactions were carried out in a volume of 10 μl containing SMUG1 excision buffer (20 mM HEPES, pH 7.9, 50 mM NaCl, 1 mM MgCl_2_, 1 mM DTT), 50 nM of fluorescein-labeled dU:dA37, dU:dG37, 5hmdU:dA37 and 5hmdU:dG containing duplex DNA and the indicated amount of SMUG1 and UV-DDB. Reactions were incubated at 37°C for each time point (up to 3 hr) and rapidly quenched by adding an equal volume of gel loading buffer (2 × formamide dye solution), heated at 95°C for 5 min, then cooled on ice for 5 min. 0.1 M NaOH was included in the quenching step to induce DNA nicking at the abasic site. The reaction product was separated by electrophoresis on 10% denaturing polyacrylamide gel and visualized using a laser scanner for fluorescence (Typhoon, Amersham). The substrate and product bands were quantified using ImageJ (NIH).

### Native PAGE

SMUG1-DNA reaction was prepared by combining 8 nM of 37 bp THF DNA with 60nM of SMUG1 in reaction buffer (20 mM HEPES, pH 7.5, 150 mM NaCl, 5 mM DTT, 0.5 mg/l BSA, and 5% glycerol) and incubated for 10 mins at RT, then were mixed with increasing amounts of UV-DDB (0-64 nM) in a final reaction volume of 10 μl. Each reaction was incubated for 30 mins at RT then immediately loaded on two pre-run 5% polyacrylamide (37.5:1, acrylamide: bis) native gels and run at 100 V for 50 min at 4°C in 1/2× TBE (4.5 mM Tris, 44.5 mM boric acid, 1 mM EDTA, pH 8.4). DNA bands were visualized using a laser scanner for fluorescence (Typhoon, Amersham). The percentage of total DNA bound by each protein was determined by measuring the band intensity present in the bound states and dividing by the total band intensity in the lane. Background signals from blank regions of the gel were subtracted from the signal intensities obtained from bands. The percentage of DNA bound in each reaction was plotted against the concentration of UV-DDB.

### DNA tightrope assay

Single-molecule DNA tightrope assay was performed as described previously (26,27). Briefly, poly-l-lysine (Wako Pure Chemicals) coated silica beads (5 μm; Polysciences Inc.) were deposited onto a PEG-treated coverslip (24 × 40 mm; Corning) in a custom flow cell. Defined lesion (abasic site) substrates were strung up across the beads via hydrodynamic flow. DNA substrates were made by tandem ligation of pSCW01 plasmid (∼2 kb) with a single, site-specific THF modification and proximal biotinylated thymine.

#### Protein labeling

Before imaging, purified His-tagged SMUG1 was labeled with secondary antibody-coated 605 nm quantum dots (Qdots; Invitrogen) through α-His primary antibody (Qiagen). For UV-DDB-facilitated dissociation experiments, quantum dot-labeled SMUG1 was injected into the flow cell at final concentrations of 2.6 nM with 1× UV-DDB conjugated to goat α-Flag primary antibody. For co-localization experiments, purified UV-DDB was conjugated to streptavidin-coated 705 nm Qdots through biotinylated goat α-Flag primary antibody (Bethyl), and quantum dot-labeled SMUG1 or UV-DDB were injected into the flow cell at final concentrations of 2.6 or 3.1 nM, respectively. We conjugated each protein in separate reaction tubes and injected them into the flow cell separately for dual-color experiments; the flow was stopped during the observation period. Furthermore, we performed a control experiment to check whether there is any unwanted interaction between 705Qdot-labeled UV-DDB (DDB1 is His-tagged) and α-His primary antibody conjugated to 605Qdots. We injected 705Qdot conjugated UV-DDB into the flow cell and then injected 605Qdot conjugated with α-His primary antibody and observed for 4 h. During this time, 20 particles were recorded, but none were co-localized. All binding experiments were carried out in tightrope buffer (25 mM HEPES, pH 7.5, 150 mM NaCl, 0.1 mg/ml BSA (Roche), 50 nM biotin and 1 mM DTT).

#### Data collection

Labeled proteins were visualized using oblique angle fluorescence (Nikon Eclipse Ti inverted microscope with Nikon 100× TIRF objective and 1.45 numerical aperture) with a 488 nm laser (power 1–2 mW) at the back focal plane to excite Qdots and the appropriate emission filter (Chroma) applied at RT. Movies were taken for 5 mins with frame rates between ∼11 and 12.5 fps.

#### Data analysis

Images were acquired using Nikon Elements (4.2) and exported as TIFF stacks for kymograph processing and analysis in ImageJ (NIH). Differences between dissociation rates and motility fractions were assessed by one-way ANOVA. The mean squared displacement (MSD) was calculated for all motile phases using custom scripts in MATLAB:


$$\begin{equation*}{\rm MSD}\ \left( {n\Delta t} \right) = \frac{1}{{N - n}}\ \mathop \sum \limits_{i\ = \ 1}^{N - n} {\left( {{x}_{i + n} - {x}_i} \right)}^2\end{equation*}$$

where *N* is the total number of frames in the phase, *n* is the number of frames at a given time step, Δ*t* is the time increment of one frame, and *x_i_* is the particle position in the *i*th frame. The diffusion coefficient (*D*) was determined by fitting a linear model of 1D diffusion to the MSD plots:


$$\begin{equation*}{\rm MSD}\ \left( {n\Delta t} \right) = \ 2D\left( {n\Delta t} \right) + y\end{equation*}$$

where *y* is a constant (*y*-intercept). Fittings resulting in *R*^2^ <0.8 or using <10% of the MSD plot were not considered.

#### SMUG1 and DDB2 recruitment after 5-hmdU treatment

U2OS cells were seeded at a density of 100,000 cells/ml on coverslips. The next day cells were transfected with SMUG1-GFP and DDB2-mCherry using lipofectamine 2000. After a 48 hr recovery period, cells were incubated with 2.5 μM EdU (Invitrogen, C10640) for 15 min and then treated for 15 min with 5 μM 5-(hydroxymethyl)-2′-deoxyuridine (5-hmdU) (Cayman Chemical, item #23381), DMSO was used for the untreated control cells. For the kinetics of SMUG1 and DDB2 recruitment, 5-hmdU was removed after 15 min, and the cells were left to recover in fresh media for 0, 15, 30, 60, 120 and 240 min. After treatment, cells were rinsed with PBS and washed with CSK extraction buffer to achieve staining of chromatin-bound proteins. The CSK buffer is composed of 100 nM NaCl, 300 mM glucose, 10 nM PIPES, 3 mM MgCl_2_, 0.5% Triton-X-100, and ddH_2_O. After the CSK extraction, cells were fixed using 4% paraformaldehyde (PFA) for 10 min. Cells were then washed three times with PBS and then permeabilized with 0.2% triton-X-100 for 10 min. The Click-IT EdU Cell Proliferation Kit (Invitrogen, C10640) was used to stain the incorporated EdU with Alexa 647 per the manufacturer's protocol. Coverslips were placed in blocking solution (10% Goat Serum, 1% BSA in PBS) for 1 hr at RT, and subsequently incubated overnight (O/N) at 4 °C with mCherry antibody (1:250 Abcam #ab167453) and GFP (1:100 Santa Cruz, #B2). The next day, after three PBS washes, cells were incubated with anti-rabbit (Alexa 594, cat# A11012) and anti-mouse (Alexa 488, cat# A21202) secondary antibodies for 1 hr at room temperature. Cells were then washed three times with PBS and then stained with DAPI at 1:5000 dilution for 10 min. Lastly, the cells were washed once with PBS and ddH_2_O and then mounted onto slides using Prolong Diamond Anti-Fade (Catalog #P36970; Molecular Probes).

Image acquisition with images having a 0.2 μm z-stack was completed using the Nikon Ti inverted fluorescence microscope. The NIS Elements software was used to deconvolute and analyze images. Within the NIS Elements analysis software, the region of interest tool was used to define cellular nuclei. DDB2-mCherry and SMUG1-GFP foci were defined with individual binary layers and the intersection feature was used to define co-localized foci. The fluorescence intensity was kept consistent for all analyzed samples. The foci counts were exported to Excel and GraphPad Prism for statistical analysis. GraphPad Prism was used to conduct an unpaired student *t*-test to compare foci formation after treatment.

#### Proximity ligation assay

U2OS cells were seeded at 1 × 10^6^ cells/ml onto a 10 cm dish. The next day cells were transiently transfected with plasmids (SMUG1-GFP, DDB2-mCherry, GFP-DDB1). Following transfection, the cells were replated at a density of 10,000 cells/well in an 8-chambered tissue culture treated glass slide (Falcon, #354118). 24 hrs later the cells were incubated with 5.0 μM 5-hmdU as detailed above. For the UV treatment, after cells were washed with PBS, a 254 nm lamp was used to expose the cells to 60 J/m^2^ UV-C through a 2 μm polycarbonate filter. (Millipore Sigma; #TTTP04700). After damage with either 5-hmdU or UV-C, cells were incubated with ice-cold CSK buffer for 2 min and then subsequently fixed with PFA and permeabilized with Triton-X as described above. After blocking for 1 hr at RT, primary antibodies to mCherry and GFP were left on the cells O/N at 4 °C, following the conditions described above. The next day cells were incubated with the PLA probes and subjected to ligation and amplification as per the manufacturer's instructions (#DUO92101). Images were acquired, deconvoluted, and analyzed as detailed above. PLA foci per nucleus was reported.

#### Western blot for PAR

U2OS cells were seeded at a density of 2 × 10^6^ cells/well in 10 cm dishes. To observe PAR in the absence of SMUG1 or DDB2. U2OS cells were transfected with 40 nM siRNA targeting SMUG1 or DDB2 respectively using Lipofectamine 2000 in antibiotic-free DMEM following the manufacturer's instructions. A non-targeting control was used for the wild-type (WT) cells. Fresh media was added 4–6 hrs post-transfection The following day, cells were replated onto a 6-well plate at 4 × 10^5^ cells/well in a 6-well plate. 48 hr post-transfection, the cells were treated with 0 μM or 5 μM 5-hmdU (Cayman Chemical, item #23381) and 0 μM or 20 μM PARGi (Sigma Aldrich, Cat # SML1781) for 15 min. After a 15 minute treatment cells were harvested for protein isolation by scraping on ice and lysed and homogenized in 4× NuPage LDS sample buffer containing a nuclease (88701, ThermoFisher). The samples were denatured for 10 min at 95°C, equal amounts were loaded on 4–12%, Bis-Tris gels (NW04120BOX), and transferred onto nitrocellulose membranes. After using Ponceau S staining to verify successful transfer, the membranes were blocked in 10% nonfat dry milk in PBST (1× phosphate-buffered saline containing 0.1% Tween 20) for 1 hr at RT and then probed for PAR, SMUG1, DDB2 and actin. Primary antibodies used: PAR (1:1000, Enzo, ALX-804-220-R100) SMUG1 (1:1000, Abcam, ab192240); DDB2 (1:1000, Abcam, ab181136). Secondary antibodies: anti-rabbit IgG (1:50,000 Sigma #A0545), or anti-mouse IgG (1:50,000 Sigma #A4416). Membranes were developed on film using SuperSignal ECL substrate (Pierce), following the manufacturer's instructions. Blots were analyzed using ImageJ siRNAs used: Control siRNA: siGENOME non-Targeting siRNA Pool #2 (Dharmacon D-001206-14-05); DDB2: 5′-AACUAGGCUGCAAGACUU-3′ Dharmacon, USA; SMUG1: Silencer siRNA (ThermoFisher, 4392420 siRNA id: s24134)

#### Immunofluorescence for PAR

U2OS cells were seeded at a density of 4 × 10^6^/dish in a 10 cm dish. The next day, cells were transfected with 40 nM of either scrambled, SMUG1, DDB2, or SMUG1/DDB2 targeting siRNA as described above. 24 hrs post transfection, the cells were re-seeded on to coverslips. The following day the cells were treated with 5-hmdU and PARGi as described above. After the 15-min treatment cells were washed with PBS and ice-cold CSK extraction buffer as described above. The cells were fixed with 1:1 methanol:acetone solution on ice for 20 min. After three PBS washes, the cells were permeabilized with 0.5% Triton-X for 10 min and put in blocking solution (see above) for 1 hr at RT. The cells were incubated with primary antibody to PAR (1:100, Enzo, ALX-804-220-R100) overnight at 4 °C in a humidity chamber. The next day the cells were washed, incubated with secondary antibody (anti-mouse, Alexa 488), stained for DAPI, and mounted as described above. Imaging and analysis were completed as explained above. Foci per nucleus was reported. Western blotting was used to confirm targeted siRNA knockdown as detailed above.

#### Seahorse flux analyzer

U2OS cells were plated on to 10 cm dishes at 1 × 10^6^/dish. The next day cells were transfected with 40nM siRNA as detailed above. 24 h post transfection the cells were replated to a 96-well plate at 6 × 10^4^/well and were left O/N in 5% CO_2_ incubator at 37°C. The next day cells were treated with 5-hmdU, PARGi, and/or PARPi (Olaparib, AZD2281, SelleckChem) in full DMEM, O/N in 5% CO_2_ incubator at 37°C. The next day the full DMEM was removed, and the cells were washed with unbuffered DMEM and incubated with 5-hmdU, PARGi, and/or PARPi in unbuffered DMEM in 0% CO_2_ incubator at 37°C for 1 hr. Basal oxygen consumption rate (OCR) and basal extracellular acidification rate (ECAR) were measured simultaneously (8 replicate wells) in real time. Oligomycin (1 μM) at injection port A, FCCP (300 nM) at injection port B, 2-DG (100 mM) at injection port C, and rotenone (1 μM) at injection port D were injected to change bioenergetic compacity and automatic measurements were captured. The basal OCR and ECAR was reported as pmol/min and mpH/min, respectively. Statistical significance was assessed through a one-way ANOVA using GraphPad Prism.

#### Cell proliferation and colony formation assays

U2OS cells were plated onto 6-well plates at a density of 300,000 cells/well. The next day cells were transfected with siRNA targeting SMUG1 or DDB2 as described above. For the cell proliferation assays, cells were replated onto a 96-well plate at a density of 3000 cells/well, 24 hrs post-transfection. Cell proliferation was assessed through the CyQUANT Direct Red Cell Proliferation assay (Invitrogen) following the manufacturer's instructions. For the colony formation assays, 24 hrs post-transfection, cells were re-plated onto 6-well plates at 500 cells/well. The next day cells were treated with 5-hmdU with continuous exposure for 8 days. On day 8, the cells were fixed with 4% PFA in PBS (15 min, RT), and colonies were stained in a 0.1% crystal violet 20% methanol solution (30 min, RT). Plates were washed with water and left to dry overnight before quantitation. Knockdown efficiency was confirmed through western blot analysis as described above.

## RESULTS

### UV-DDB shows specific binding affinity to DNA duplexes containing uracil (U) and 5-hydroxymethyluracil (5-hmdU)

**Figure 1.**
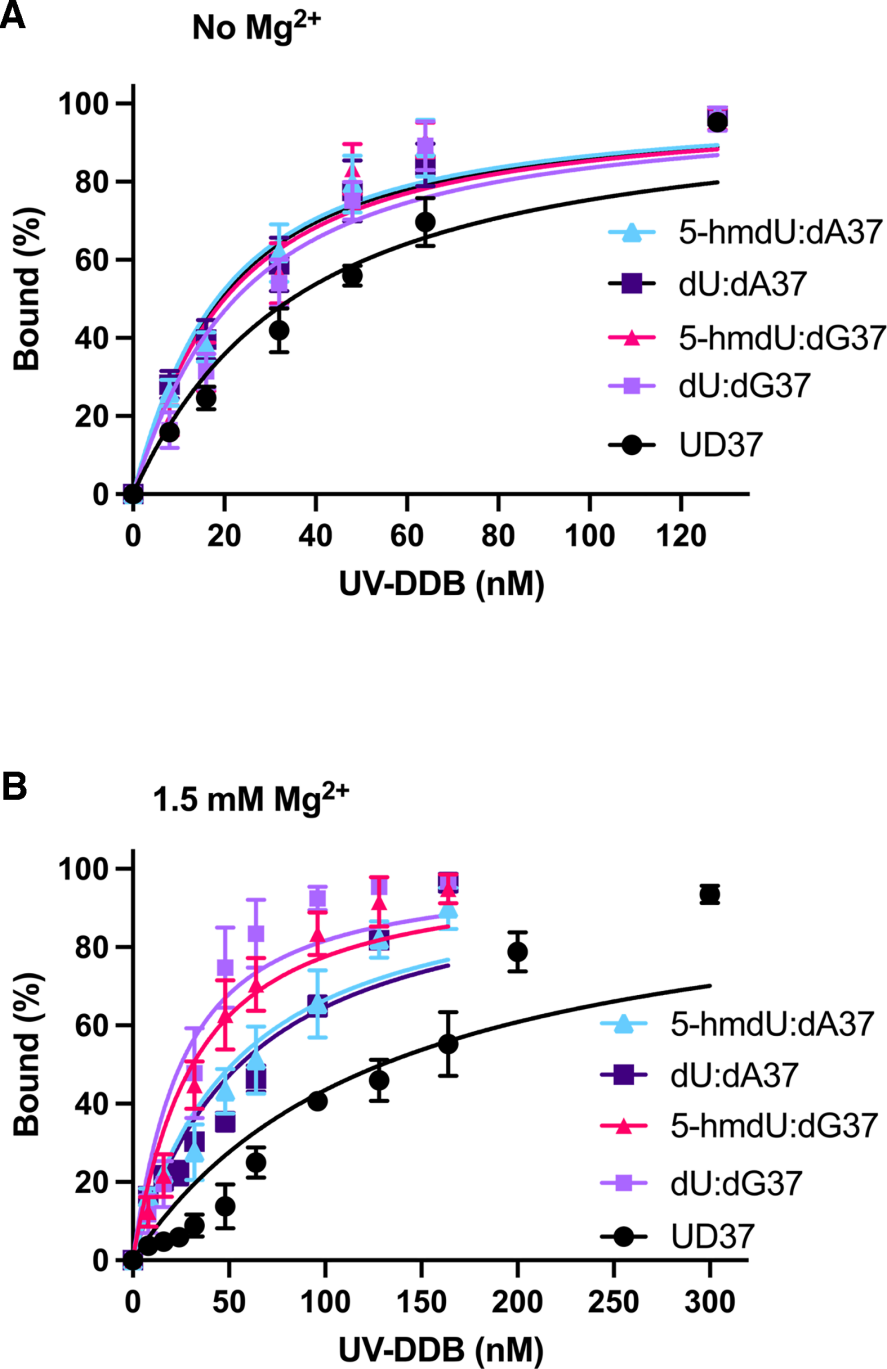
UV-DDB binding specifically to uridine and 5-hydroxymethyl uridine. (**A**) UV-DDB binding isotherms for 5-hmdU:dA37, dU:dA37, 5-hmdU:dG37, dU:dG37 and undamaged DNA (UD37) in the absence of magnesium. Data is plotted as mean ± SEM. of three independent experiments. (**B**) UV-DDB binding isotherms for 5-hmdU:dA37, dU:dA37, 5-hmdU:dG37, dU:dG37 and undamaged DNA (UD37) in the presence of 1.5 mM magnesium. Data is plotted as mean ± SEM. of three independent experiments.

UV-DDB can bind avidly to abasic sites and 8-oxoG (26). We therefore first sought to quantify UV-DDB binding affinities for undamaged DNA and four different types of DNA damage: dU paired with adenine or guanine and 5-hmdU paired with adenine or guanine embedded in a 37 bp duplex. Electrophoretic mobility shift assays (EMSA) were performed in the absence and presence of magnesium (1.5 mM), in the reaction buffer, running buffer, and gel, as we have found magnesium can increase damage specificity by decreasing non-specific DNA affinity (Figure 1A and B, Supplementary Figure S11b–k) (31). Apparent equilibrium dissociation constants (*K*_d_) for UV-DDB binding were determined from the binding isotherms and are presented in Table 1, along with other apparent *K*_d_ for other substrates. Our data clearly demonstrated that UV-DDB, in the absence of magnesium, bound to DNA containing a dU or 5-hmdU moiety with around a 2-fold higher affinity than that for undamaged DNA. Interestingly, UV-DDB, in the presence of magnesium (1.5 mM) had an approximately 5-6 fold increase in specificity for mispaired dU:dG and 5-hmdU:dG compared with that for undamaged DNA. Importantly, these results indicate that UV-DDB has a higher specificity for dU:dG and 5-hmdU:dG pairs as compared to dU or 5-hmdU paired with A, which are all greater than the affinity UV-DDB has for undamaged DNA.

**Table 1.** Magnesium increasing binding specificity of UV-DDB, related to Figure 1

Substrate	No Mg^2+^	1.5 mM Mg^2+^	Fold difference
37 bp duplex	*K*_d_^1^ (nM)	*K*_d_^1^ (nM)	
dU:dA37	15 ± 1.0	52 ± 3.4	3.5
dU:dG37	18 ± 1.6	21 ± 2.1	1.1
5-hmdU:dA37	14 ± 1.1	48 ± 3.0	3.3
5-hmdU:dG37	16 ± 1.5	27 ± 1.9	1.7
THF^2^	0.4 ± 0.2	ND	
CPD^2^	5 ± 0.3	ND	
8-oxoG:C^2^	35 ± 2.1	ND	
G:T^3^	8 ± 0.8	ND	
undamaged	31 ± 2.0	125 ± 9.1	4.0

^1^Apparent equilibrium dissociation constant (*K*_d_) were determined from data presented in Figure 1A and B. These data are based on three independent experiments analyzed on two gels for each experiment. The binding isotherm was fit to a quadratic equation to obtain an apparent *K*_d_. The reported *K*_d_ values are the mean of three independent experiments ± the SEM of the fit to the observed data.

^2^Beecher *et al.*, DNA Repair 2020 (31).

^3^Beecher and Van Houten, unpublished.

### UV-DDB stimulates SMUG1 excision activity

**Figure 2.**
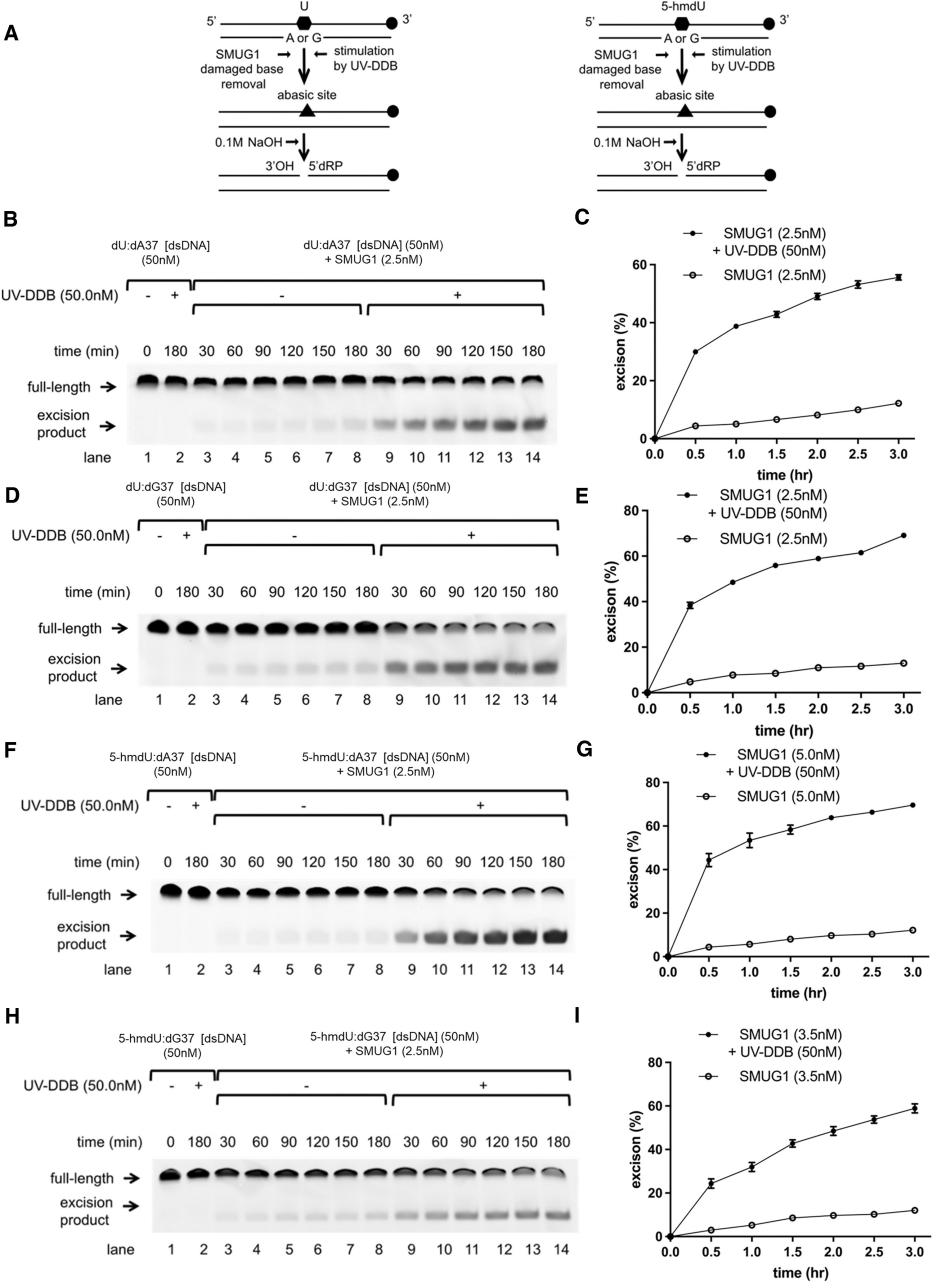
UV-DDB stimulates SMUG1 on uridine containing DNA. (**A**) Schematic representation of the DNA substrate containing dU:dA or dU:dG pair (left) or 5-hmdU:dA or 5-hmdU:dG pair (right) and the proposed reaction scheme. (B, D, F, H) Stimulation of SMUG1 excision kinetics by UV-DDB. SMUG1 (2.5 nM) was incubated with dsDNA (50 nM) containing dU:dA pair (**B**), dU:dG pair (**D**), 5-hmdU:dA pair (**F**), 5-hmdU:dG pair (**H**) in the absence (−) or presence (+) of UV-DDB (50 nM) at 37°C. Aliquots were withdrawn at each time point and analyzed on a 10% denaturing polyacrylamide gel. Positions of the un-cleaved full-length substrate and excised product are indicated by arrows. (**C**, **E**, **G**, **I**) Quantification of the stimulation of SMUG1 excision kinetics by UV-DDB in (B, D, F and H, respectively). Excision product formation was quantified using ImageJ software. The excision percentage was plotted as mean ± SEM from three independent experiments, each run on duplicate gels.

We then examined whether UV-DDB can stimulate the enzymatic activity of SMUG1. To determine the effect of UV-DDB on SMUG1-catalyzed dU and 5-hmdU base excision paired with A or G, a limiting amount of SMUG1 (2.5–5.0 nM) that produced about 5% excision alone was incubated with dU:dA37, dU:dG37, 5-hmdU:dA37 and 5-hmdU:dG37 duplex oligonucleotide (50 nM) in the absence or the presence of UV-DDB up to 3 hr. In this assay, the SMUG1-processed DNA was treated with NaOH to convert AP sites to nicks before denaturing PAGE. We also discovered that stimulation of SMUG1 excision activity by UV-DDB was concentration-dependent, reaching a maximum at 50 nM, then declining at higher concentrations (Supplementary Figure S2). A four to five-fold stimulation of SMUG1 activity was observed in the presence of UV-DDB (50 nM). Purified UV-DDB by itself showed no detectable DNA glycosylase activity (lane 2 at Figure 2B, D, G and I). These excision kinetic data indicate that UV-DDB may stimulate the turnover and activity of SMUG1 or facilitate product release during BER. This concept was investigated in more detail using native gel electrophoresis and the single molecule tightrope assay, described below.

### UV-DDB facilitates dissociation of SMUG1 from abasic DNA

Our previous studies (26,27) have suggested that UV-DDB facilitates the dissociation of OGG1, MUTYH and APE1 molecules on THF (tetrahydrofuran, abasic site analog) containing DNA thus increasing product release rates which may contribute increasing their rates of turnover. UV-DDB binds abasic sites with an apparent equilibrium dissociation constant *K*_d_, of 0.4 nM (Table 1) and we found that SMUG1 binds to abasic sites ∼160-fold less tight, with an apparent *K*_d_ of 65 ± 9.8 nM (Supplementary Figure S3A and B). We thus sought to investigate whether UV-DDB could displace SMUG1 from abasic sites using native PAGE. SMUG1 (60 nM) -THF37 duplex DNA (8 nM) complex was pre-formed by incubating these two components for 10mins at room temperature (RT). Upon incubation of SMUG1 with oligonucleotide, >80% of the DNA was in the form of a complex with SMUG1 (Supplementary Figure S3C, lane 8). Then increasing amount of UV-DDB (0–64 nM) was added in the absence of SMUG1 (lanes 1–7) or the presence of SMUG1 (lanes 8–14). After 30 mins incubation at RT, UV-DDB occupies the abasic site after dissociating SMUG1 from its abasic site product to prevent product reassociation allowing recycling of SMUG1, and therefore inducing stimulation of SMUG1 to find another substrate. No co-complexes were observed.

**Figure 3.**
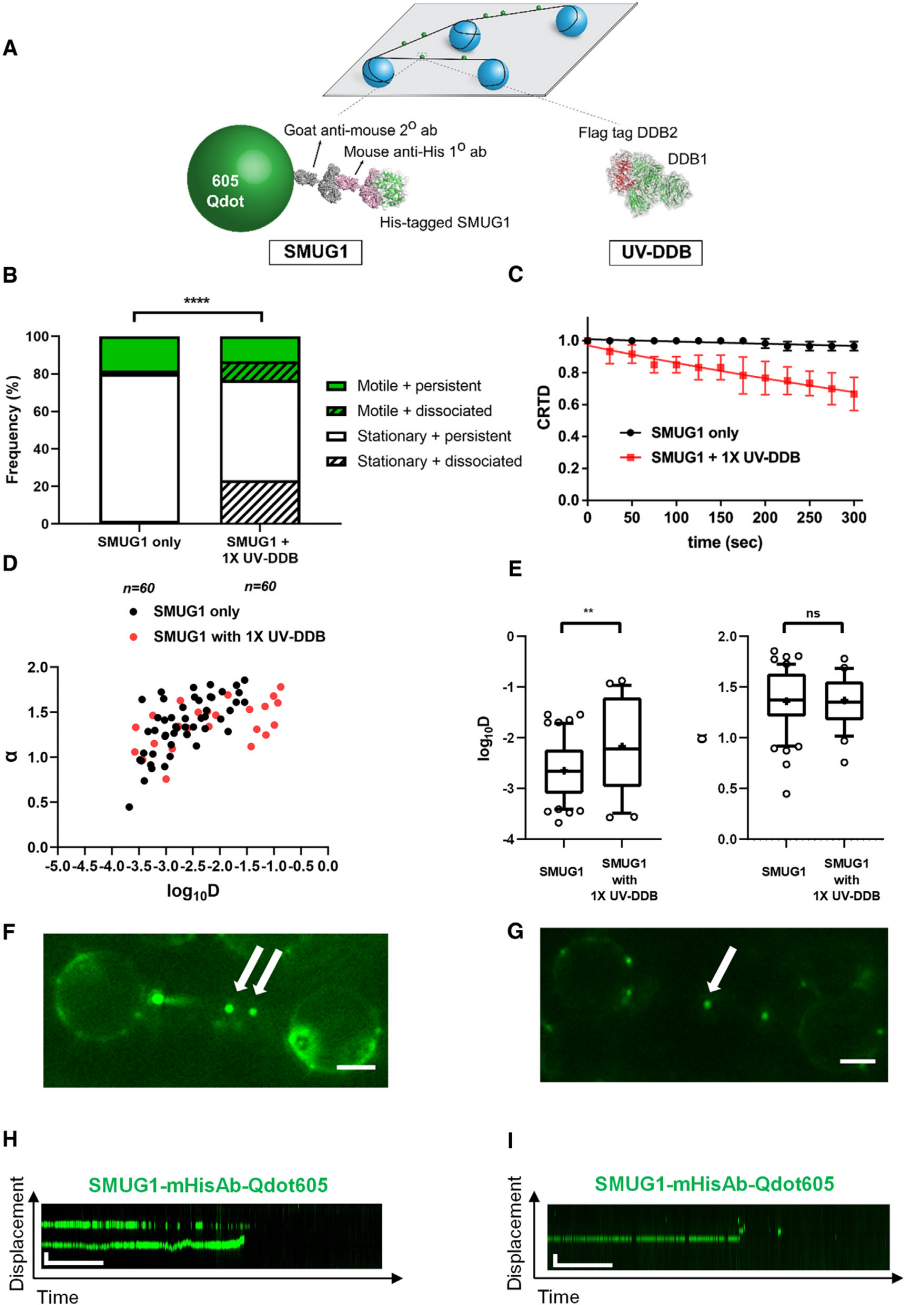
Single-molecule analysis reveals that UV-DDB stimulates SMUG1 by facilitated dissociation. (**A**) Experiments design of DNA tightrope assay to study UV-DDB induced dissociation of SMUG1. (top) Long DNA substrates with defined abasic sites (THF) every 2 kb are suspended between silica beads (bottom, left) His-tagged SMUG1 is labeled with primary mouse-anti-His antibody and secondary goat-anti-mouse antibody conjugated to a 605 nm Qdot, (bottom, right) unlabeled UV-DDB. (**B**) Stacked bar graph showing the fraction of motile (green) versus stationary (white) and persistent (solid) versus dissociating (diagonal lines) particles of 605 Qdot labeled SMUG1 in the absence (–) or presence (+) of unlabeled 1× UV-DDB on abasic (THF) DNA during 300s observation. (****, *P* < 0.0001 by χ2 test). (**C**) Effects of UV-DDB on the binding lifetimes of SMUG1–DNA complexes. Data plotted as the mean ± SEM from three independent experiments. For each condition, CRTD (cumulative residence time distribution) is fit to a single exponential decay function to obtain the half-life. (**D**) Anomalous diffusion exponent (α) versus diffusion coefficient (log_10_*D*) plotted for SMUG1 (black-filled circles) and SMUG1 with 1× UV-DDB (red-filled circles). (**E**) Box and whisker plot (10–90 percentile) of left, the Anomalous diffusion exponent (α) and right, the diffusion coefficient (log_10_*D*) calculated for SMUG1 only (*n* = 50 particles) and SMUG1 with 1× UV-DDB (*n* = 24 particles) phases on long DNA substrates with defined abasic sites (THF) every 2 kb. +, sample mean, ** *P* < 0.01, ns, non-specific by two-tailed Student's *t*-test (**F**) Image of 605 Qdot-labeled SMUG1 (green) on abasic (THF) tightrope suspended between beads in the 1× presence of unlabeled UV-DDB. Scale bar represents 2.5 μm. Arrows point to dissociated SMUG1 particles. Horizontal and vertical scale bars represent 50 s and 2 kb, respectively. (**G**) Image of 605Q dot-labeled SMUG1 (green) on abasic (THF) tightrope suspended between beads in the 1× presence of unlabeled UV-DDB. Scale bar represents 2.5 μm. Arrow points to the dissociated SMUG1 particle. (**H**) Kymograph of 605 Qdot-labeled SMUG1 (green) with non-motile or motile dissociated particles (F). (**I**) Kymograph of 605 Qdot-labeled SMUG1 (green) with dissociated particle (G). Horizontal and vertical scale bars represent 50 s and 2 kb, respectively.

We also tested this hypothesis that unlabeled UV-DDB may increase the rate of Qdot-labeled SMUG1 dissociation from abasic (THF) sites at the single-molecule level. SMUG1 was labeled with 605 nm quantum dots (Qdots) using an antibody sandwich approach in which a primary mouse anti-His antibody was bound to a His-tagged SMUG1 protein, then goat anti-mouse secondary antibody-coated 605 nm Qdots (Figure 3A). Qdot-labeled SMUG1 was observed in the absence or presence of unlabeled UV-DDB on DNA tightropes containing one abasic (THF) site every 2 kb (Methods) for a total of 5 min, and the number of Qdot-labeled proteins dissociating during that time was noted. SMUG1 displayed three-fold increase in the frequency of dissociation (during a 5-min observation window) when the same amount of UV-DDB was added (Figure 3B). Addition of equimolar of UV-DDB to SMUG1 decreased the half-life of SMUG1 from 4950 s to 580 s, as shown by a decreasing cumulative resonance time distribution (CRTD) (Figure 3C). These data thus indicate that UV-DDB binding to abasic sites may help facilitated dissociation of SMUG1 bound to abasic sites. The presence of UV-DDB was also found to cause non-motile SMUG1 molecules to undergo diffusion on DNA and dissociate, Figures 3F–I, S4 and corresponding Supplementary videos 1 and 2. Diffusion behavior of each motile SMUG1 molecule was further analyzed by characterization of its anomalous diffusion exponent (α factor) and diffusion coefficient (*D*) in Figure 3D and E. The mean α factor of SMUG1 only and SMUG1 + 1× UV-DDB is 1.36 ± 0.31 and 1.37 ± 0.25, and its diffusion coefficient is (2.65 ± 0.58) × 10^−2^ μm^2^/s and (2.17 ± 0.93) × 10^−2^μm^2^/s, respectively. Compared to free SMUG1, we found that addition of 1× UV-DDB showed significant increase on diffusion coefficient (*D*) of motile SMUG1 molecules.

### UV-DDB can co-localize with SMUG1 on DNA containing abasic sites

**Figure 4.**
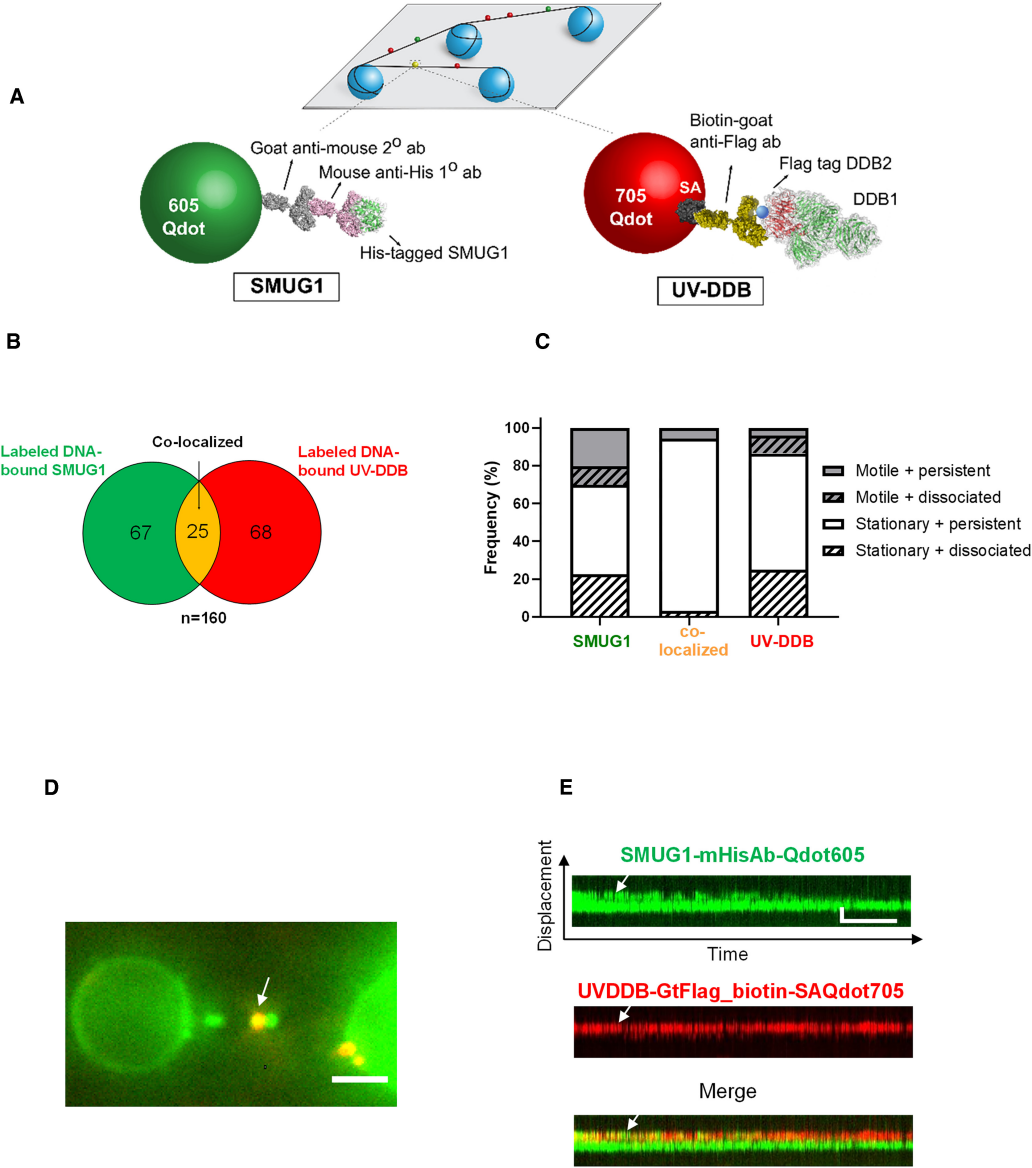
Single-molecule co-localization of UV-DDB with SMUG1 on abasic DNA tightropes. (**A**) Schematic of the DNA tightrope assay. Long DNA substrates with abasic sites every 2 kb were suspended between 5 μm poly-L-lysine coated silica beads. Anti-His primary antibody was used to link the His-tagged SMUG1 to the 605 Qdot. Biotin conjugated anti-Flag primary antibody was used to link Flag-tagged UV-DDB to streptavidin-coated 705 Qdot. Uniquely labeled SMUG1 and UV-DDB were observed interacting on abasic DNA tightropes in real-time and their behavior and frequency of co-localization were recorded. (**B**) Venn diagram showing the number of proteins that co-localized (yellow) on abasic (THF) tightropes or were observed separately for 605 Qdot-labeled SMUG1 (green) with 705 Qdot-labeled UV-DDB (red) in the dual-color assay. (**C**) Stacked bar graph showing the fraction of motile (gray) versus stationary (white) and persistent (solid) versus dissociating (diagonal lines). Results obtained with individual and co-localized particles. (SMUG1; SMUG1 behavior in the presence of UV-DDB, Co-local; co-localized SMUG1/UV-DDB behavior, UV-DDB; UV-DDB behavior in the presence of SMUG1). Data is re-plotted as a sub-set of (B). (**D**) Image of co-localized (yellow) Qdot-labeled SMUG1 (green) and UV-DDB (red) on abasic (THF) tightrope suspended between beads. The scale bar represents 2.5 μm. Arrow points to co-localized particles. (**E**) Kymograph of co-localized SMUG1 and UV-DDB. Top, SMUG1 (green); middle, UV-DDB (red); bottom, merged (yellow). Horizontal and vertical scale bars represent 50 s and 2 kb, respectively.

Since both SMUG1 and UV-DDB are capable of binding to abasic sites individually, we sought to further investigate their potential interactions on the DNA containing abasic sites. To do this, we used an orthogonal labeling strategy for direct dual-color fluorescence imaging of His-tagged SMUG1, labeled with 605 nm Qdots and Flag-tagged UV-DDB, labeled a biotinylated goat anti-Flag primary antibody and streptavidin-coated 705 nm Qdots, as described above (Figure 4A). Control experiments indicated that there is no exchange of Qdots between SMUG1 and UV-DDB (data not shown).

Qdot-labeled SMUG1 was first injected into a flow cell containing DNA tightropes with one abasic site (THF) every 2 kb, and then Qdot labeled UV-DDB was injected. After injection, all flow was stopped. Owing to the transient nature (mobility and dissociation) of SMUG1 and UV-DDB binding to damaged DNA, we reasoned that co-localization might be a relatively rare event. We found that co-localization of SMUG1 and UV-DDB accounted for 15.6% of all particles (Figure 4B). Some of these co-localized molecules (Figure 4C–E) were found to diffuse on the DNA together, suggesting direct interactions on DNA; however, the overall motion and dissociation of the repair proteins did not change substantially when complexed together (Figure 4C). The co-localized particles were classified as stationary and persistent 91.1% of the time, indicating they were bound to an abasic site. Through the kymograph analysis, we were able to show motion of the co-localized particles on the DNA together (Supplementary Figure S5B). The kymographs of merged channels (Figure 4E) showed co-localization of green and red signals, indicating specific binding of SMUG1 with UV-DDB (additional kymographs shown in Supplementary Figure S5 and Supplementary video 3). Also note that the mobility and rate of dissociation of SMUG1 is altered by UV-DDB. Taken together, these data suggest that UV-DDB can associate with and migrate together with SMUG1 on DNA.

### Co-localization of UV-DDB and SMUG1 in cells treated with 5-hmdU

**Figure 5.**
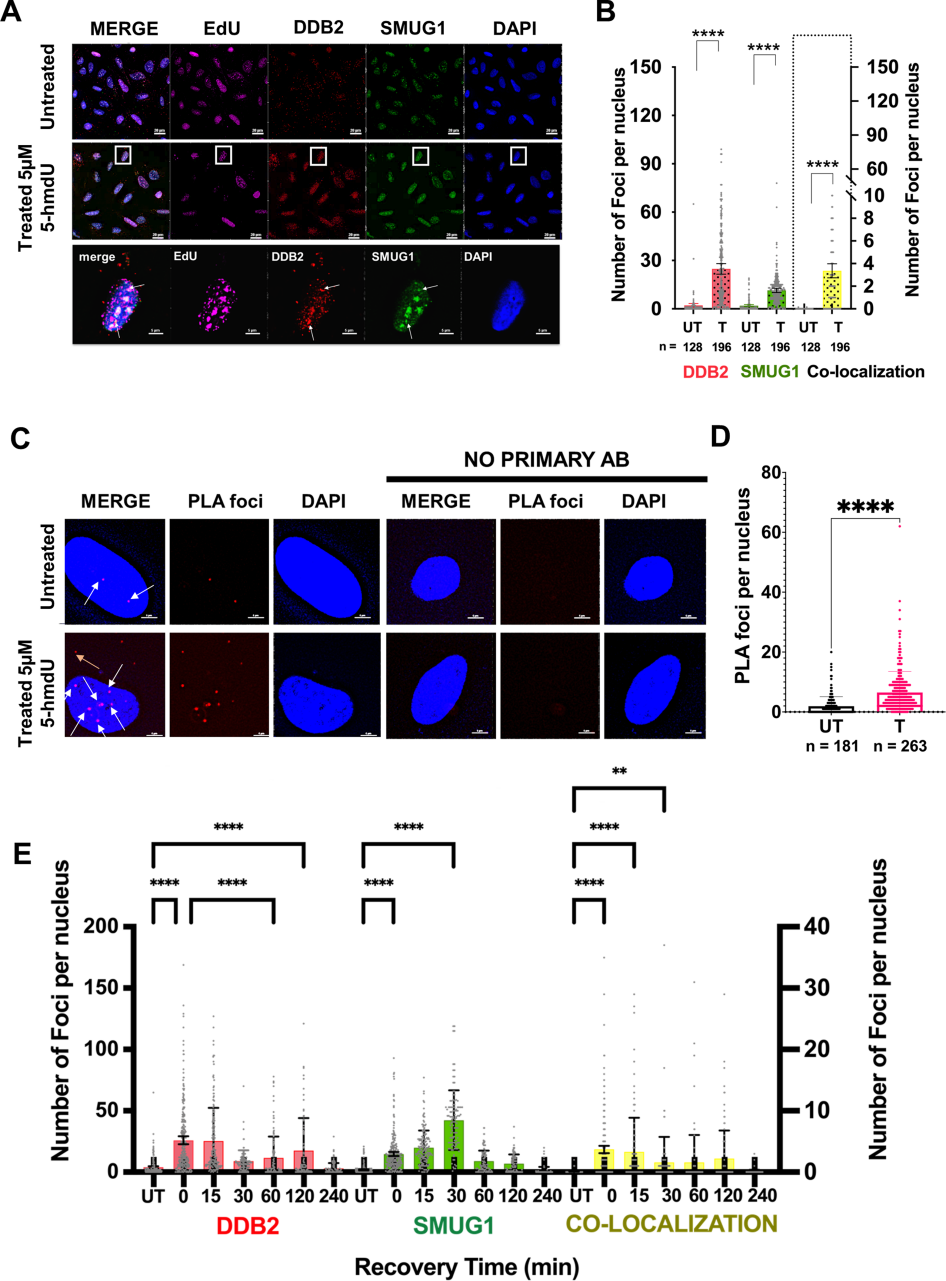
DDB2 and SMUG1 co-localize in cells after treatment with 5-hmdU. (**A**) Immunofluorescence images demonstrating co-recruitment of DDB2-mCherry and SMUG1-GFP after labeling with 2.5 μM EdU and 15-minute treatment with 5.0 μM 5-hmdU. White arrow pointing towards co-localized foci. Scale bar 5 μm. (**B**) Bar graph representing quantification of A (**** *P* < 0.0001; unpaired *t*-test). (**C**) Proximity ligation assay demonstrating co-recruitment of DDB2-mCherry and SMUG1-GFP after 15-min treatment with 5.0 μM 5-hmdU. White arrows point towards nuclear PLA foci; yellow arrow is cytoplasmic PLA foci. Scale bar 5 μm. (**D**) Bar graph representing quantification of (C) (**** *P* < 0.0001; unpaired *t*-test). (**E**) Quantitation of the kinetics of DDB2-mCherry and SMUG1-GFP recruitment after a 15-min treatment with 5-hmdU over the course of 4 h (*** *P* < 0.001, **** *P* < 0.0001; one way-ANOVA). *N* = 100–250 cells scored per time point.

We previously demonstrated that UV-DDB is recruited to sites of 8-oxoG lesions and co-localizes with OGG1 in cells (26,29). To this end, we sought to determine if UV-DDB and SMUG1 are recruited to sites of 5-hmdU incorporation with cells. 5-hmdU is a nucleoside analog that gets tri-phosphorylated and incorporated into the DNA during replication, which occurs during the S-phase of the cell cycle. U2OS cells transiently expressing SMUG1-GFP and DDB2-mCherry were treated with 5-hmdU (5.0 μM) for 15 min and then collected for immunofluorescence staining (Figure 5A and B). EdU labeling was utilized to distinguish S-phase positive cells. We observed a significant increase (*P* < 0.0001, Student's t-test) in the number of SMUG1 (4.5-fold) and DDB2 (11-fold) foci after 5-hmdU treatment compared to control cells, as well as a significant increase in their co-localization (Figure 5B). To confirm there was no competition for incorporation between the nucleoside analogs, EdU and 5-hmdU, we looked at EdU labeling in both the presence and absence of 5-hmdU, and we observed no difference in EdU positive cells (Supplementary Figure S6A), which was approximately 50% EdU positive. First, we utilized a proximity ligation assay (PLA) as a positive control to show an interaction between the two subunits of UV-DDB, GFP-DDB1 and DDB2-mCherry, after UV-damage, and were able to show an increase in PLA signal after damage (Supplementary Figure S6B). To validate an interaction between SMUG1 and DDB2 in cells, we performed a proximity ligation assay (PLA). Antibodies to GFP and mCherry were used to assess interactions between SMUG1 and DDB2, respectively. We were able to show an increase in nuclear PLA foci after treatment with 5-hmdU (Figure 5C and 5D). Additionally, we found an increase in PLA signal outside the nucleus, which will be further analyzed in future studies. These experiments demonstrate UV-DDB and SMUG1 both work on the repair of 5-hmdU lesions in cells. We next sought to observe the kinetics of DDB2-mCherry and SMUG1-GFP recruitment over the course of four hours after a 15-min treatment with 5-hmdU (5.0 μM). Through immunofluorescence staining we observed robust recruitment of DDB2 immediately after treatment with 5-hmdU that peaked prior to peak recruitment of SMUG1, which occurs 30 min post-treatment. (Figure 5E, Supplementary Figure S6E). Interestingly we observed a biphasic recruitment of DDB2 post 5-hmdU treatment, an early recruitment which peaked at 15 min post-treatment, and a later resurgence at 120 min post-treatment (Figure 5E, Supplementary Figure S6E). The second wave of DDB2 recruitment could be indicative of roles for UV-DDB in the downstream BER pathway.

**Figure 6.**
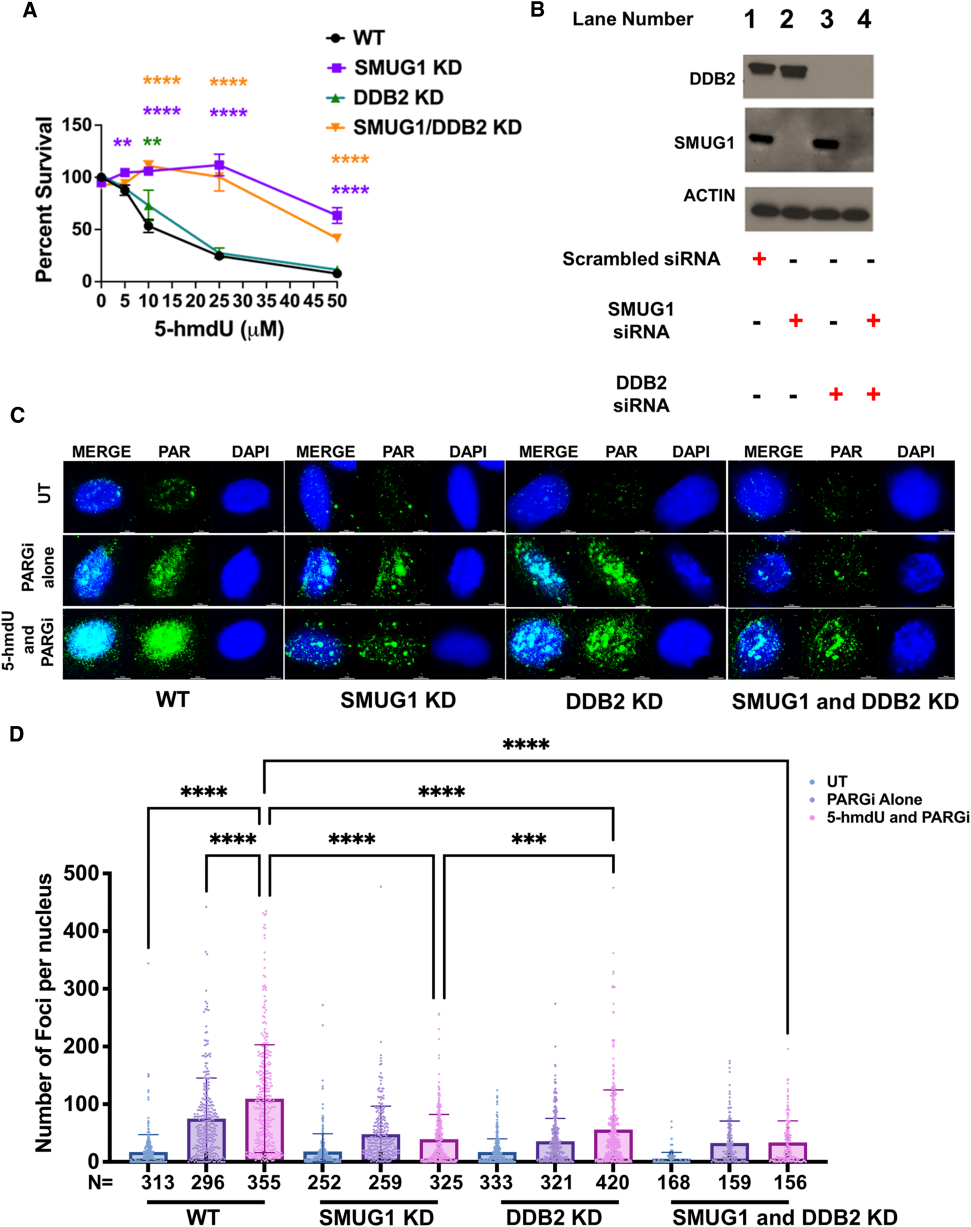
Active repair of 5-hmdU results in cell toxicity driven by PARP1 activation. (**A**) Cell survival curves of U2OS cells transfected with either scrambled, SMUG1, DDB2, SMUG1/DDB2 targeting siRNA and treated with a range of concentrations of 5-hmdU, continuously for 8 days. Data represent mean ± SEM from three independent experiments, each performed in triplicate. (**p < 0.01; **** p < 0.0001; by two-way ANOVA). (**B**) Western blot analysis to confirm knockdown of SMUG1 and DDB2 after 8-day cell survival study, actin was used as loading control. (**C**) Immunofluorescence images demonstrating PAR expression in U2OS cells (WT, and after siRNA knockdown of SMUG1 or DDB2 treated with 5.0 μM 5-hmdU and 20 μM PARGi for 15 min, untreated controls received equal concentration DMSO. Images are representative of three biological experiments. Scale bar 5 μm (**D**) Bar graph representing quantification of (C) (*** *P* < 0.001, ****P < 0.0001; one-way ANOVA).

To further elucidate the roles of SMUG1 and DDB2 during 5-hmdU repair, we measured cell sensitivity to 5-hmdU in cells depleted of SMUG1 or DDB2. Consistent with the literature (16) the loss of SMUG1 prevented toxicity to 5-hmdU in U2OS cells (Figure 6A, B, Supplementary Figure S7A). Supporting the idea that it is the repair of 5-hmdU that creates toxicity in cells, not the incorporation of this nucleoside analog. We next examined whether DDB2 depletion influences cell sensitivity to 5-hmdU. Unlike the loss of toxicity when SMUG1 was knocked-down, toxicity was observed in the absence DDB2. Whereas the double knock-down of both SMUG1 and DDB2 showed reduced 5-hmdU toxicity. These results suggest that once SMUG1 has processed the lesion, DDB2 is required to help turnover SMUG1 from the toxic abasic site intermediate (16). SMUG1’s creation of an abasic site, is further processed by APE1 resulting in a single strand break (SSB) in the phosphodiester backbone. The SSB is then bound by the ADP-ribosyltransferase, PARP1, resulting in its activation to produce poly (ADP)-ribose (PAR) for the recruitment of downstream BER repair proteins (2). The PAR is rapidly catabolized by poly(ADP-ribose) glycohydrolase (PARG), so in order to observe PAR accumulation a PARG inhibitor is required (32). Key intermediates formed in BER include the abasic site created after the glycosylase removes the damaged base and the SSB created when the abasic site is processed (33,34). To further validate that 5-hmdU treatment initiated BER, PAR accumulation was monitored by immunofluorescence (Figure 6) and western blot analysis (Supplementary Figure S7B). U2OS cells treated with 5-hmdU (5.0 μM, 15 min) and a PARGi (20 μM) resulted in robust production of PAR (Figure 6C and D, Supplementary Figure S7B, lane 3) To test further test that SMUG1 and DDB2 work together to help drive the repair of 5-hmdU, we used siRNA targeting SMUG1 or DDB2 and looked at PAR accumulation after 5-hmdU and PARGi treatment. Using immunofluorescence, the knockdown of SMUG1 significantly reduced PAR accumulation after 5-hmdU treatment (Figure 6C and D). Loss of DDB2 significantly reduced PAR accumulation compared to WT cells, however, there was still some PAR accumulation as BER was still being initiated by SMUG1 (Figure 6C and D). Our findings are consistent with previous studies showing that PARylation is dependent upon SMUG1 and formyl-dU (35). Moreover, the loss of DDB2 leads to a decrease in PAR accumulation after 5-hmdU treatment, supporting the hypothesis that DDB2 functions during BER processing of 5-hmdU.

**Figure 7.**
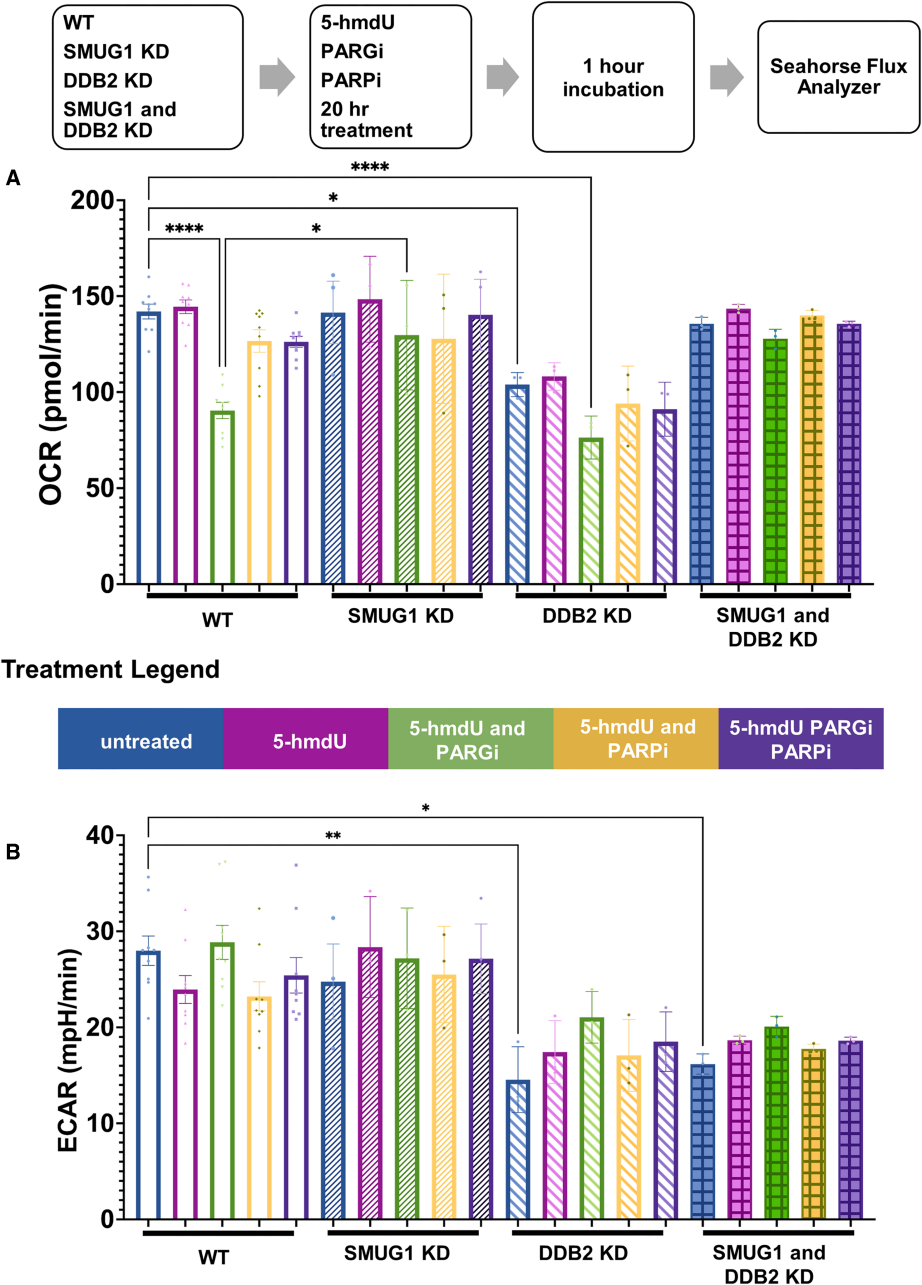
Cellular bioenergetics during active 5-hmdU repair. (**A**) Basal oxygen consumption rate of U2OS WT, SMUG1-KD, DDB2-KD, SMUG1/DDB2-KD cells treated with 5-hmdU and PARGi. Untreated cells received DMSO control. (**B**) Basal extracellular acidification rate of U2OS WT, SMUG1-KD, DDB2-KD, SMUG1/DDB2-KD cells treated with 5-hmdU and PARGi. Untreated cells received DMSO control. Data represent mean ± SEM from 3-9 independent experiments performed with 6 repeats/experiment for 3 independent readings over time. (* *P* < 0.05, ** *P* < 0.01, **** *P* < 0.0001; one-way ANOVA).

We next sought to determine whether 5-hmdU treatment-induced PARP1 hyperactivation alters bioenergetics of WT cells in cells depleted of SMUG1, DDB2, or SMUG1/DDB2. It has been previously shown that PARP1 hyperactivation after certain forms of DNA damage, leads to a decrease in oxidative phosphorylation (36,37). Extracellular Flux analysis was used to assess basal oxygen consumption (OCR) rates or extracellular acidification rates (ECAR) after 5-hmdU and PARGi treatment, using a Seahorse instrument. We observed a robust decrease in the OCR of WT U2OS cells treated with 5-hmdU and PARGi (Figure 7A). This reduction of basal OCR is not observed in WT cells treated with the PARP1 inhibitor (PARP1i), Olaparib, and PARGi, or in cells deficient in SMUG1 (Figure 7A). Interestingly, in the absence of 5-hmdU treatment, there is a lower basal OCR in cells deficient in DDB2 (Figure 7A). ECAR, a measure of glycolysis indicated that loss of DDB2 caused a significant decrease in ECAR in the absence of cell treatments. 5-hmdU treatment in the presence of PARP1i and/or PARGi had no impact in ECAR, Figure 7B. Since cellular bioenergetics is due in part to NAD levels, these results are consistent with the concept that intermediates from SMUG1-initiated repair of 5-hmdU may lead to PARP1 hyperactivation, causing a decrease in oxidative phosphorylation. Taken together, these results support the cooperativity between SMUG1 and DDB2 during 5-hmdU repair in cells.

## DISCUSSION

Monofunctional glycosylases, such as SMUG1 are product inhibited, binding more avidly to the abasic site created compared to the target lesion, reviewed in (38). Additionally lesions in the context of nucleosomes can be difficult for some glycosylases to recognize (24). DNA repair needs to progress in a timely manner to avoid the accumulation of potentially toxic intermediates. There is growing evidence of cooperativity between NER and BER, and to this end our lab previously demonstrated roles for UV-DDB in the processing of 8-oxoG by stimulating the activities of OGG1, MUTYH and AAG/MPG (26–29). We show in this present study using biochemical, single-molecule, and cell biology approaches that product inhibition of SMUG1 binding to abasic sites can be overcome through the actions of UV-DDB. Electrophoretic mobility shift assays demonstrated UV-DDB can bind to SMUG1 target substrates and can stimulate SMUG1 activity 4-5-fold. Using the technically innovative DNA tightrope assay we were able to show UV-DDB stimulates SMUG1 turnover through a facilitated dissociation mechanism. Lastly, we were able to show co-localization and transient interaction of UV-DDB and SMUG1 in cells treated with 5-hmdU. Together these studies revealed that SMUG1 excision of a novel oxidative lesion 5-hmdU, and it's subsequent turnover, like OGG1, MUTYH and AAG/MPG is stimulated by UV-DDB.

Many groups have shown that DNA glycosylases have difficulty acting on base damage embedded in a nucleosome (39–47). For example, Delany and coworkers found that SMUG1 works very inefficiently on U:G mispairs embedded in nucleosomes regardless of their position (24). During nucleotide excision repair, UV-DDB as part of the CRL^DDB2^ E3 ubiquitin ligase has been shown to ubiquitinate H2A, H3, and H4 in nucleosomes containing UV-induced photoproducts, reviewed in (48). This activity is believed to allow the nucleosome to become more unstable and allow access by subsequent repair factors. Polo and coworkers have shown that DDB2 can directly mediate chromatin decompaction (49). Additionally, Thoma and coworkers have shown that UV-DDB can alter the register of a lesion on a nucleosome by as many as three base pairs (50). Thus, DDB2 alone or as part of the CRL^DDB2^ ligase could be important in allowing access to specific types of base damage, including 8-oxoG or 5-hmdU within nucleosomes. To this end, we have shown that the arrival of DDB2 at sites of 8-oxoG damage precedes OGG1 arrival (26). Recently we demonstrated that DDB2 binding to 8-oxoG was necessary and sufficient to mediate changes to chromatin structure at the telomeres (29). It is still unknown if DDB2 alone or as part of the DDB1-Cul4A-E3-ligase is required for the recruitment of other chromatin remodelers to the damage site. Consistent with these findings our data demonstrate that maximal recruitment of DDB2 occurs immediately after 5-hmdU damage (15-minute treatment), whereas SMUG1 reaches peak recruitment 30 min post damage (Figure 5).

**Figure 8.**
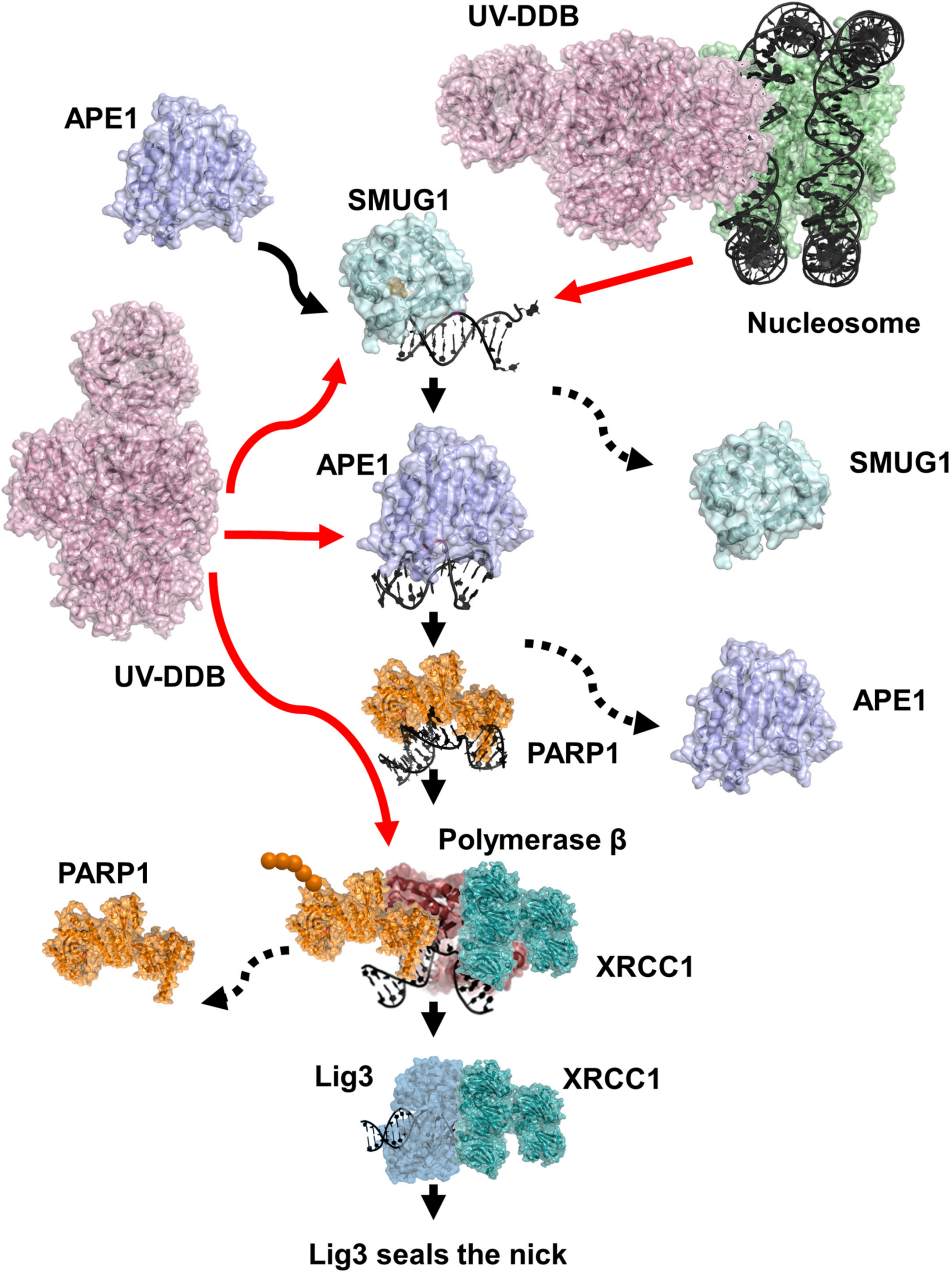
Working model for UV-DDB’s role in SMUG1 mediated removal of 5-hmdU during BER pathway. Schematic representation of the proposed BER pathway including UV-DDB. Red arrows indicate roles of UV-DDB in the pathway. Dashed arrows represent protein dissociation. UV-DDB appears to be rapidly recruited to damaged sites in chromatin and helps facilitate the processing of 5-hmdU by SMUG1. Biochemical and single molecule data suggest that UV-DDB transiently associates with SMUG1 at abasic sites to increase its turnover and stimulate BER, and cellular data supports transient interactions between UV-DDB and SMUG1 (see text for description).

Based on all the experimental data presented here and from the literature, Figure 8 illustrates a working model for UV-DDB and SMUG1 in BER. We believe that rapid recruitment of UV-DDB to lesion sites in chromatin is necessary to facilitate the BER of 5-hmdU by SMUG1. Furthermore, due to the high-affinity UV-DDB has for abasic sites, UV-DDB works to promote SMUG1 turnover from abasic sites to progress repair. We have previously demonstrated that UV-DDB can facilitate APE1 turnover from abasic sites and stimulate APE1 activity in the context of 8-oxoG repair (26). Data presented here are consistent with our previous studies demonstrating UV-DDB stimulates glycosylases through a facilitated dissociation mechanism (26–28,51,52). This facilitated dissociation mechanism is driven by competition for the abasic site, analogous to a baton passing event, where UV-DDB grabs the abasic site from SMUG1, facilitating SMUG1 turnover and subsequent downstream BER events. Future studies will investigate the role of UV-DDB in stimulating SMUG1 activity in the context of nucleosomes as it has been previously shown that SMUG1 has difficulties working when the lesion is embedded in nucleosomes (24). In addition, future biochemical and cellular experiments will be necessary to better understand how UV-DDB acts as a damage sensor to cooperate with OGG1, MUTYH, SMUG1 and other glycosylases to help mediate BER.

BER needs to proceed in a concerted and timely manner to avoid toxic intermediate formation. The role of toxic intermediates during BER has been the topic of several studies (16,53–55). Work from the Neuburger lab demonstrated that *Smug1*^−/−^ MEFs are resistant to 5-hmdU treatment, implying the repair of 5-hmdU that is toxic, not the lesion itself (16). Key intermediates formed during BER are abasic sites and single-strand breaks (SSB) which can lead to polymerase stalling and blocks to replication if left unrepaired (34,54). During BER, the SSBs are bound by PARP1, which uses NAD to autoPARylate to signal the recruitment of downstream repair factors (32,35). As shown in Figure 6 we observed robust production of PAR after treating cells with 5-hmdU and a PARG inhibitor, which is lost when 5-hmdU repair is not initiated by SMUG1 and reduced when cells are depleted of DDB2. We also demonstrated in Figure 7 a change in cellular bioenergetics during active repair of 5-hmdU that provides explanations for the nature of the cell toxicity. Surprisingly, knockdown of DDB2 caused lower amount of oxidative phosphorylation and glycolysis. Future studies will focus on elucidating whether the mechanism of cell death is associated with a bioenergetic collapse, driven by PARP1 hyperactivation (33), and the mechanism by which depletion of DDB2 causes a decrease in bioenergetics. In addition, more studies need to be conducted to understand if UV-DDB regulates PARP1 activity. Additionally in the absence of SMUG1, UNG may provide some backup function in the repair of 5-hmdU, the mechanism of which needs further study (16). SMUG1 has been shown to play multiple roles in genome stability. Fugger and coworkers illustrated a role for SMUG1 in contributing to synthetic lethality in homologous recombination deficient cells during the repair of 5-hmdU (53). Moreover, the Ciccia group provided a working model that in BRCA1/2 deficient cells gap accumulation and subsequent mutagenesis are driven by SMUG1 activity followed by PRIMPOL-mediated fork repriming and translesion synthesis by REV1-polζ (55). Taken together our group has shown that UV-DDB has a consistent but unique role in BER by stimulating the activities of OGG1, MUTYH, APE1, AAG/MPG, and now SMUG1 (26–29). Future experiments studying UNG and other mammalian glycosylases will be necessary to show the extent to which UV-DDB functions as a damage sensor to help process lesions during BER.

## DATA AVAILABILTY

All raw data is available and can be released upon request from the corresponding author, vanhoutenb@upmc.edu.

## Supplementary Material

gkad206_Supplemental_Files
