# Supplementary material for: UV-DDB stimulates the activity of SMUG1 during base excision repair of 5-hydroxymethyl-2'-deoxyuridine moieties
Source: Nucleic Acids Res. 2023 Mar 27;51(10):4881–98. doi: 10.1093/nar/gkad206 (PMC10250209; doi:10.1093/nar/gkad206)
Supplement: gkad206_Supplemental_Files [file gkad206_supplemental_files.zip › Supplementary Data_03_01_2023_FIN.pdf]

Supplemental Data

UV-DDB stimulates the activity of SMUG1 during base excision repair of 5-hydroxymethyl-2'-deoxyuridine moieties

Sunbok Jang[1,3,4*], Sripriya J. Raja[1,2*], Vera Roginskaya[1,4], Matthew A. Schaich [1,4], Simon C. Watkins[5], and Bennett Van Houten[1,2,4#]

[1]UPMC Hillman Cancer Center, University of Pittsburgh, Pittsburgh, PA 15213, USA

[2]Molecular Pharmacology Graduate Program, School of Medicine, University of Pittsburgh, Pittsburgh, PA 15213 USA.

[3]College of Pharmacy, Graduate School of Pharmaceutical Sciences, Ewha Womans University, Seoul, 03760, Republic of Korea.

[4]Department of Pharmacology and Chemical Biology, School of Medicine, University of Pittsburgh, Pittsburgh, PA 15213, USA

[5] Center for Biologic Imaging, University of Pittsburgh, Pittsburgh, PA 15261, USA.

* These two authors contributed equally to the manuscript and share first authorship.

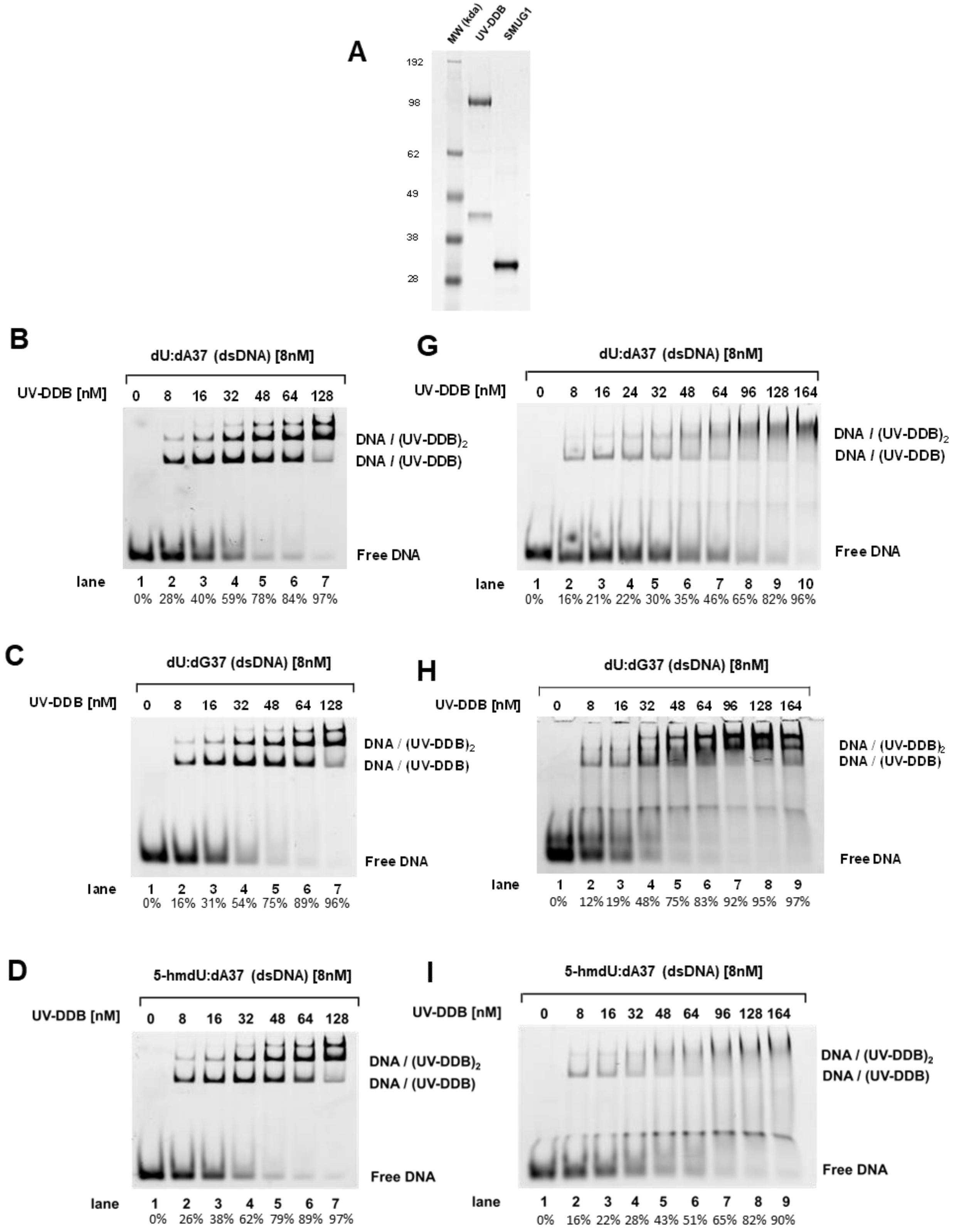

A
MW (kda)
UV-DDB
SMUG1
192
98
62
49
38
28
B
dU:dA37 (dsDNA) [8nM]
UV-DDB [nM] 0 8 16 32 48 64 128
DNA / (UV-DDB)2
DNA / (UV-DDB)
Free DNA
lane 1 2 3 4 5 6 7
0% 28% 40% 59% 78% 84% 97%
C
dU:dG37 (dsDNA) [8nM]
UV-DDB [nM] 0 8 16 32 48 64 128
DNA / (UV-DDB)2
DNA / (UV-DDB)
Free DNA
lane 1 2 3 4 5 6 7
0% 16% 31% 54% 75% 89% 96%
D
5-hmdU:dA37 (dsDNA) [8nM]
UV-DDB [nM] 0 8 16 32 48 64 128
DNA / (UV-DDB)2
DNA / (UV-DDB)
Free DNA
lane 1 2 3 4 5 6 7
0% 26% 38% 62% 79% 89% 97%
G
dU:dA37 (dsDNA) [8nM]
UV-DDB [nM] 0 8 16 24 32 48 64 96 128 164
DNA / (UV-DDB)2
DNA / (UV-DDB)
Free DNA
lane 1 2 3 4 5 6 7 8 9 10
0% 16% 21% 22% 30% 35% 46% 65% 82% 96%
H
dU:dG37 (dsDNA) [8nM]
UV-DDB [nM] 0 8 16 32 48 64 96 128 164
DNA / (UV-DDB)2
DNA / (UV-DDB)
Free DNA
lane 1 2 3 4 5 6 7 8 9
0% 12% 19% 48% 75% 83% 92% 95% 97%
I
5-hmdU:dA37 (dsDNA) [8nM]
UV-DDB [nM] 0 8 16 32 48 64 96 128 164
DNA / (UV-DDB)2
DNA / (UV-DDB)
Free DNA
lane 1 2 3 4 5 6 7 8 9
0% 16% 22% 28% 43% 51% 65% 82% 90%

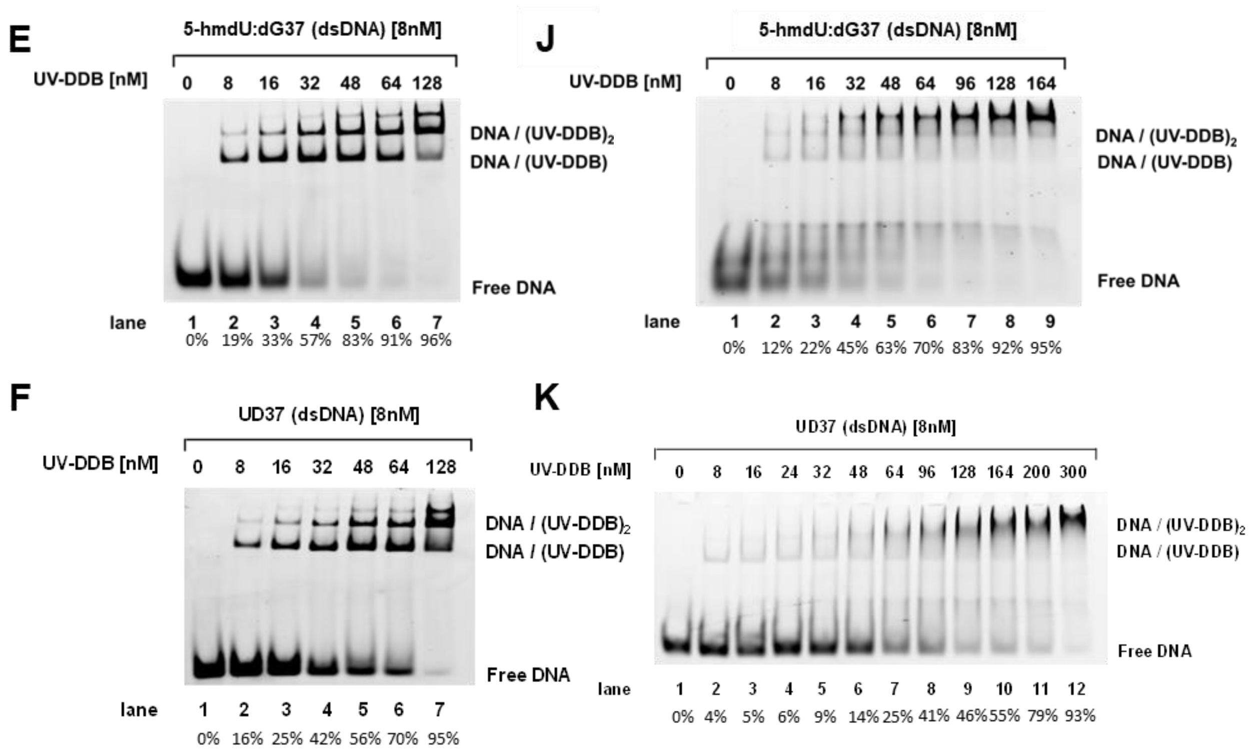

Figure S1 (related to Fig 1). Purified proteins and UV-DDB EMSA experiments.

(A) Coomassie stain of SDS-PAGE showing purified proteins used in this study. **(B-K)** Representative native gels for EMSA experiments, with average percentage of DNA bound at each concentration indicated (as calculated by the intensity of free DNA band compared to all other bands). B-F in the absence of magnesium; G-K in the presence of magnesium UV-DDB was mixed with different 37 bp fluorescein-labelled dsDNA substrates with different lesions/modifications: dU:dA37, dU:dG37, 5-hmdU:dA37, 5-hmdU:dG37 and undamaged. (U: Uracil, 5-hmdU: 5-(hydroxymethyl)deoxyuracil, UD: Undamaged).

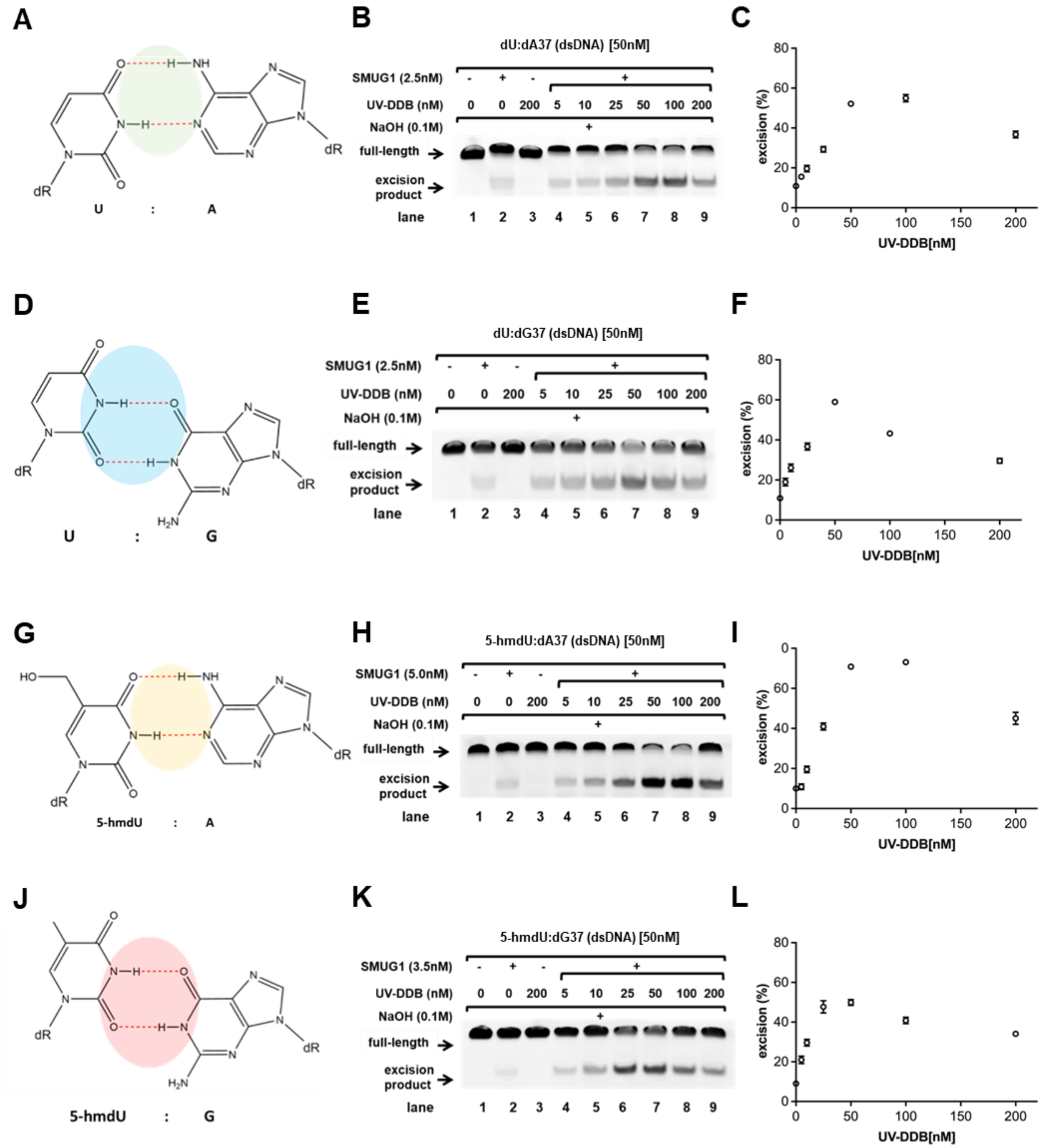

Figure S2 (related to Fig 2). Substrate chemical structure and SMUG1 activities in the absence or presence of UV-DDB.

(A) Chemical structure of uracil paired with adenine. **(B)** Effect of UV-DDB concentration on stimulation of SMUG1 excision. dU:dA37 dsDNA was incubated with SMUG1 and/or increasing amounts of UV-DDB at 37°C for 2hrs and separated by denaturing polyacrylamide electrophoresis. **(C)** Quantification of (B). Percent of total DNA that was excised by SMUG1

plotted as a function of UV-DDB concentration. data shown as the mean of three experiments ± SEM **(D)** Chemical structure of uracil paired with guanine. **(E)** Effect of UV-DDB concentration on stimulation of SMUG1 excision. dU:dG37 dsDNA was incubated with SMUG1 and/or increasing amounts of UV-DDB at 37°C for 2hrs and separated by denaturing polyacrylamide electrophoresis. **(F)** Quantification of (E). Percent of total DNA that was excised by SMUG1 plotted as a function of UV-DDB concentration. data shown as the mean of three experiments ± SEM. **(G)** Chemical structure of 5-(hydroxymethyl)deoxyuracil paired with adenine. **(H)** Effect of UV-DDB concentration on stimulation of SMUG1 excision. 5-hmdU:dA37 dsDNA was incubated with SMUG1 and/or increasing amounts of UV-DDB at 37°C for 2 hrs and separated by denaturing polyacrylamide electrophoresis. **(I)** Quantification of (H). Percent of total DNA that was excised by SMUG1 plotted as a function of UV-DDB concentration. data shown as the mean of three experiments ± SEM. **(J)** Chemical structure of 5-(hydroxymethyl)deoxyuracil paired with guanine. **(K)** Effect of UV-DDB concentration on stimulation of SMUG1 excision. 5-hmdU:dG37 dsDNA was incubated with SMUG1 and/or increasing amounts of UV-DDB at 37°C for 2 hrs and separated by denaturing polyacrylamide electrophoresis. **(L)** Quantification of (K). Percent of total DNA that was excised by SMUG1 plotted as a function of UV-DDB concentration. data shown as the mean of three experiments ± SEM.

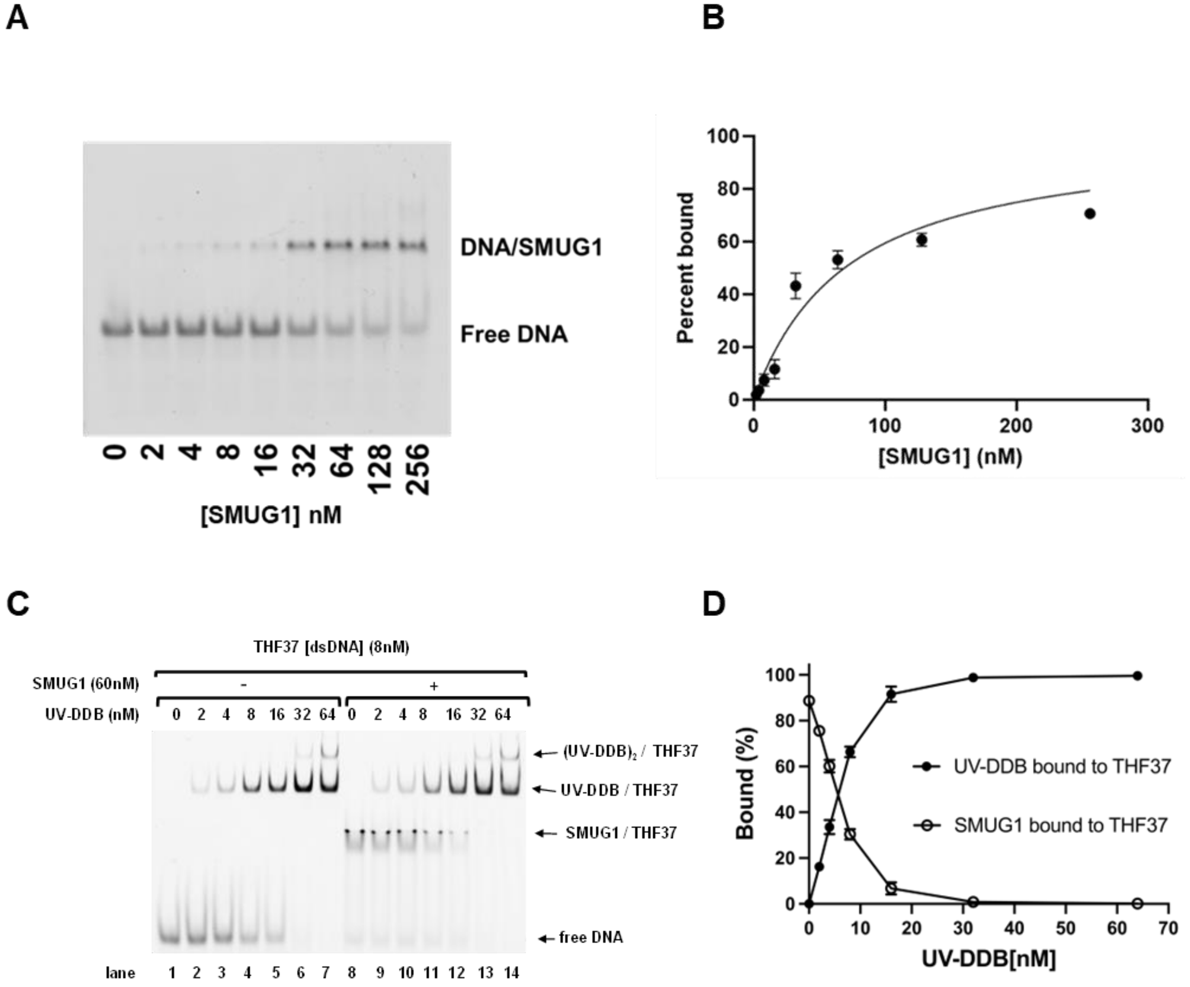

Figure S3 (related to Fig 3). UV-DDB displacement of SMUG1 from abasic DNA and SMUG1 activity on abasic DNA

EMSAs were utilized to determine the binding affinity of SMUG1 to the THF37 substrate. **(A)** A representative EMSA gel, DNA and DNA:protein complexes labeled. **(B)** Quantification and fit of the EMSA data to the equation in the materials and methods. These data were generated from three replicates quantified on two gels each, with standard errors shown as error bars. The resultant K_d was 65 ± 9.8 nM. **(C)** Displacement of SMUG1 on abasic sites by UV-DDB, shown by EMSA. Binding reactions of THF37 and increasing amounts of UVDDB with or without SMUG1 were separated by native PAGE. Protein-DNA complexes were identified based on band migration and labelled accordingly. Representative gel shown, N=3. **(D)** Quantification of (C) from lane 8 to 14. Bound percent of total DNA by UV-DDB or SMUG1 are plotted as a function of UV-DDB concentration. Data shown as the mean of two experiments ± SEM.

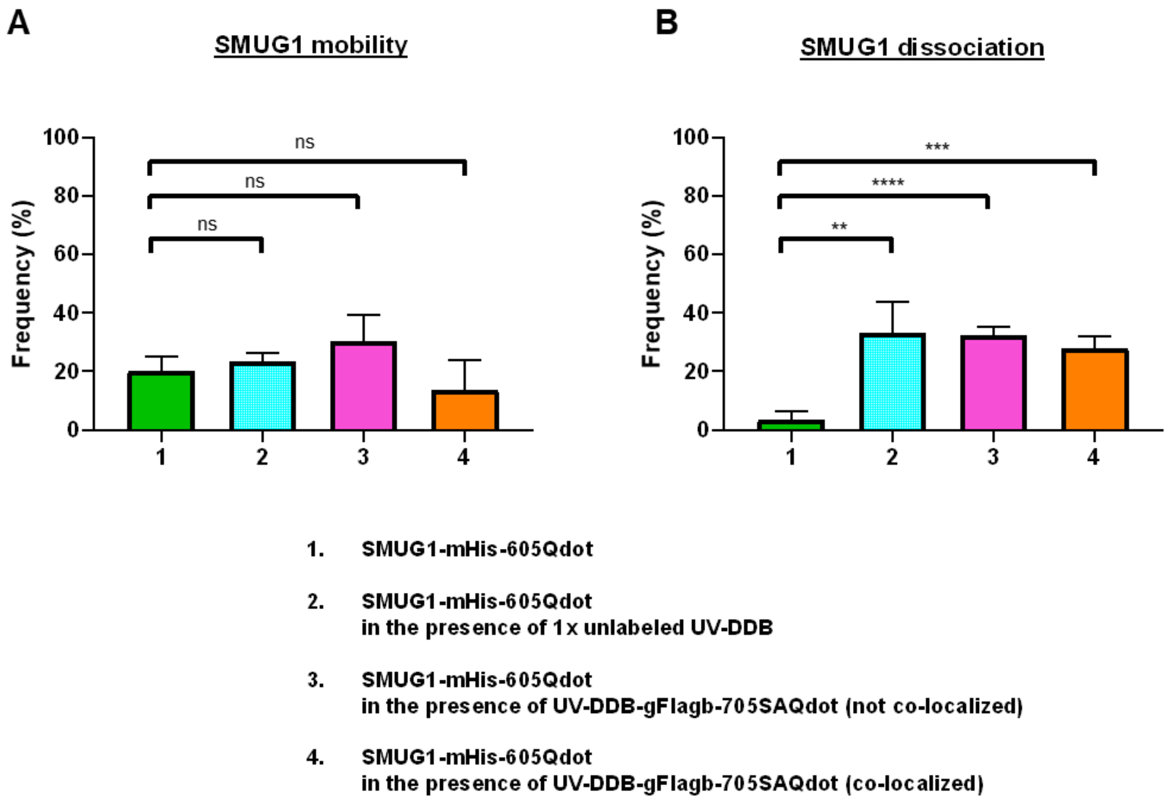

Figure S4 (related to Fig 3 & 4). Single molecule analysis reveals UV-DDB stimulates turnover of SMUG1 by facilitated mobility and dissociation.

(A) Mobility of 605 Qdot-labeled SMUG1 on DNA tightropes containing abasic sites (THF) in the absence (1 ,n=60) or presence (2, n=60, 3, n=67 & 4, n=25) of UV-DDB. Bar graph data shown as means ± SD with three (1,2) or four (3,4) independent experiments. (ns; not significant by two-tailed Student's t test). **(B)** Dissociation of 605 Qdot-labeled SMUG1 on DNA tightropes containing abasic sites (THF) in the absence (1,n=60) or presence (2, , n=60, 3, n=67 & 4, n=25) of UV-DDB. Bar graph data shown as means ± SD with three (1,2) or four (3,4) independent experiments. (** $p<0.01$, *** $p<0.001$, **** $P<0.0001$ by two-tailed Student's t test).

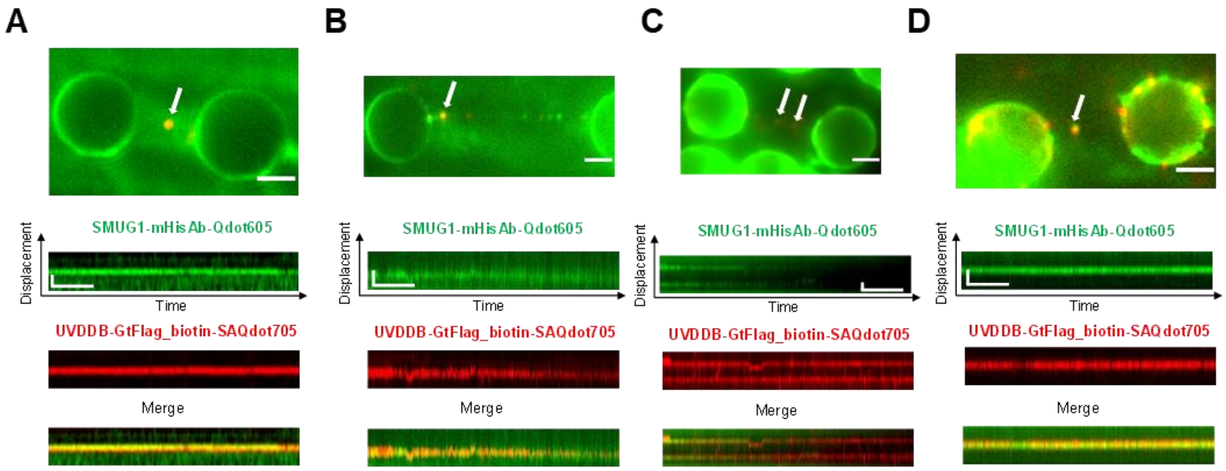

Figure S5. DNA tightrope assay showing co-localization of UV-DDB and SMUG1 (related to Fig 4).

(A-D) Additional still frames and corresponding kymographs of co-localized SMUG1 and UV-DDB (SMUG1: green, UV-DDB: red, and merge: yellow). Top, scale bar represents 2.5 µm; arrows point to co-localized particles. Bottom, horizontal, and vertical scale bars represent 50 s and 2 kb, respectively.

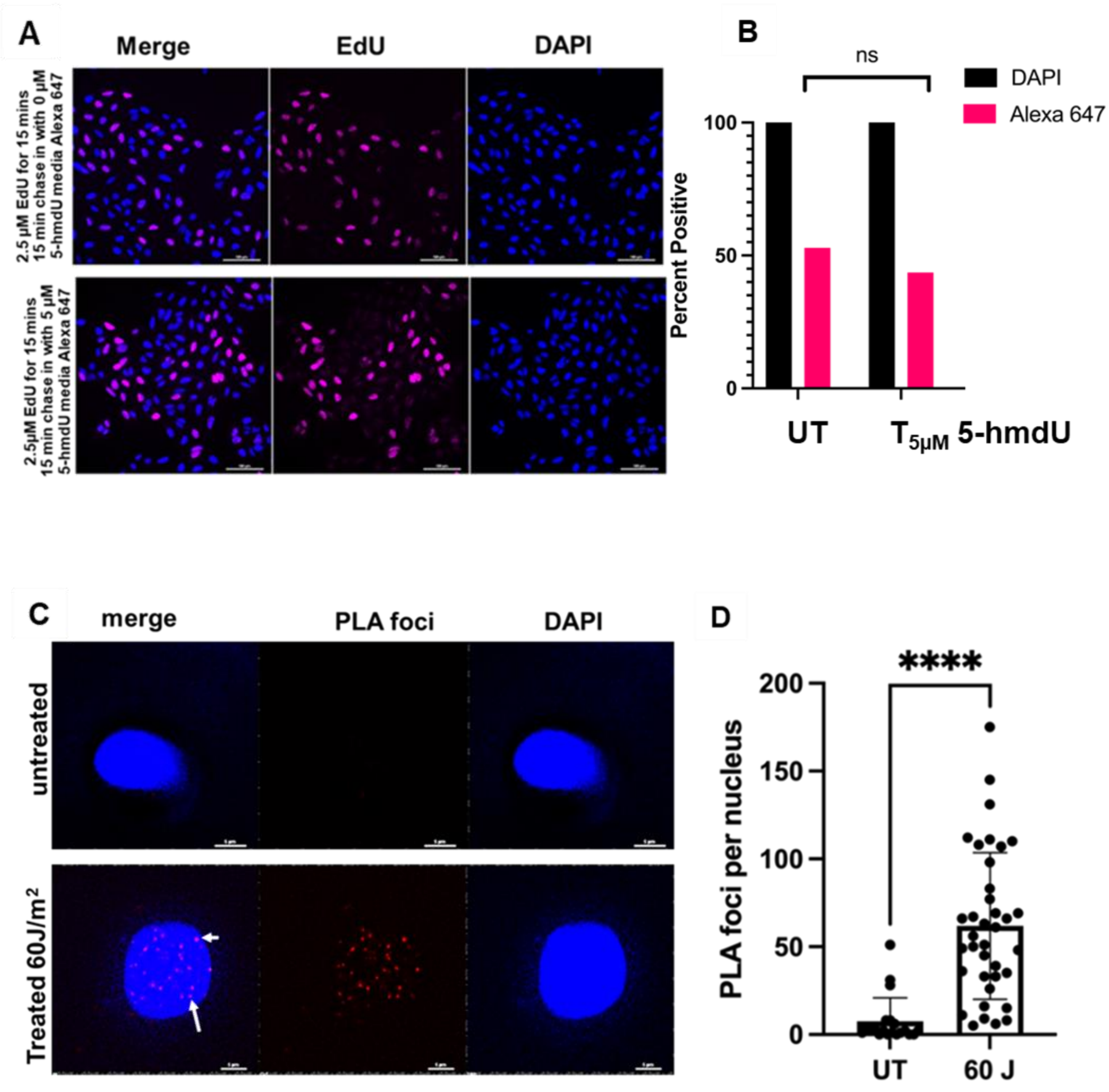

Panel E on next page

E

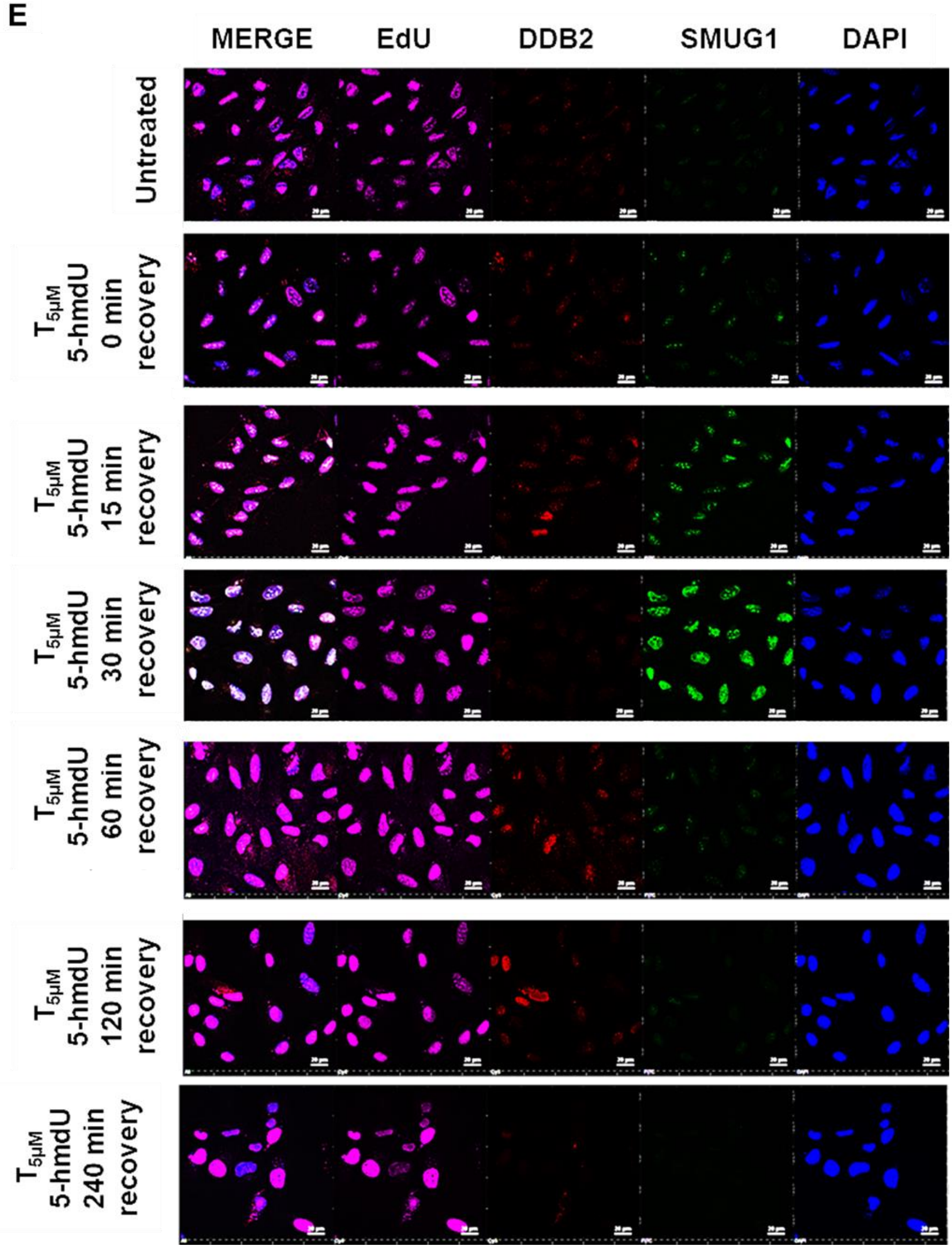
MERGE
EdU
DDB2
SMUG1
DAPI
Untreated
T5μM 5-hmdU 0 min recovery
T5μM 5-hmdU 15 min recovery
T5μM 5-hmdU 30 min recovery
T5μM 5-hmdU 60 min recovery
T5μM 5-hmdU 120 min recovery
T5μM 5-hmdU 240 min recovery

Figure S6 Cellular experiments to study the role of UV-DDB and SMUG1 in 5-hmdU repair in cells. (Related to Fig 5): (A and B) Quantitation of EdU positive cells in the presence and absence of 5-hmdU. **(C and D)** Proximity ligation assay for GFP-DDB1 and DDB2-mCherry after UV-damage.(**** $p<0.0001$; unpaired t-test) **(E)** Representative images for the kinetics of SMUG1 and DDB2 recruitment shown in figure 5.

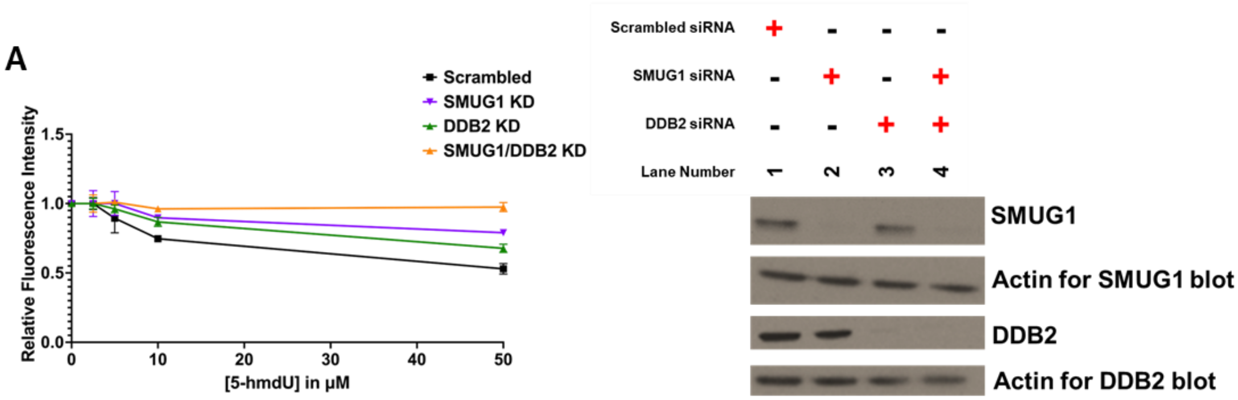

Two Way ANOVA Results				
	2.5μM	5.0μM	10μM	50μM
SMUG1 KD vs. Scrambled	ns	ns	0.0053	<0.0001
SMUG1 KD vs. DDB2 KD	ns	ns	ns	ns
SMUG1 KD vs. SMUG1/DDB2 KD	ns	ns	ns	0.0003
Scrambled vs. DDB2 KD	ns	ns	ns	0.0070
Scrambled vs. SMUG1/DDB2 KD	ns	ns	<0.0001	<0.0001
DDB2 KD vs. SMUG1/DDB2 KD	ns	ns	ns	<0.0001

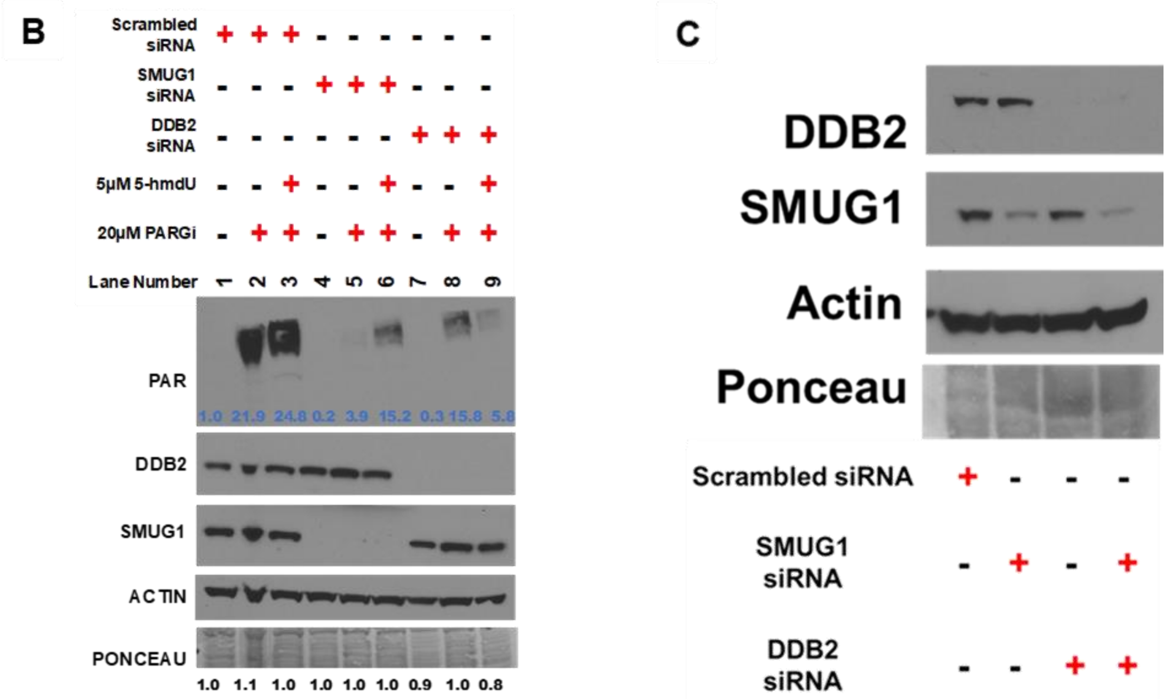

Figure S7 5-hmdU repair is toxic in cells. (Related to Fig 6): (A) Cell survival after 48-hour treatment with 5-hmdU in U2OS cells after siRNA knockdown of SMUG1, DDB2, SMUG1/DDB2. A non-targeting, scrambled control was used. The CyQUANT direct red cell proliferation assay was used to measure cell survival. Representative plot of four biological replicates. Knockdown

was confirmed through western blot and a two-way ANOVA was used to assess statistical significance. **(B)** Western blot for PAR expression in U2OS cells (WT, and after siRNA knockdown of SMUG1 or DDB2 treated with 5.0 μM 5-hmdU and 20 μM PARGi for 15 min, untreated controls received equal concentration DMSO**. (C)** Western blot confirming knockdown of SMUG1, DDB2, SMUG1/DDB2 after 48 hours for PAR IFs shown in Figure 6.

Descriptions of Videos

Video 1: 605 nm-Qdot labelled-SMUG1 induced motion on a DNA tightrope containing abasic sites in the presence of unlabeled UV-DDB corresponding to Figure 3F. Data were collected at 11.38 fps and are played back at 30 fps.

Video 2: 605 nm-Qdot labelled-SMUG1 induced motion and dissociation from a DNA tightrope containing abasic sites induced by unlabeled UV-DDB corresponding to Figure 3G. Data were collected at 11.38 fps and are played back at 30 fps.

Video 3: Transient co-localization and displacement of 605 nm-Qdot labeled-SMUG1 with 705 nm-Qdot labeled-UV-DDB. Middle complex of both proteins (arrow) on the DNA tightrope containing abasic sites and a non-motile SMUG1 molecule (on right), corresponding to Figure 4D. Data were collected at 1.74 fps and are played back at 12 fps.
